# Metal Chelation as a Powerful Strategy to Probe Cellular Circuitry Governing Fungal Drug Resistance and Morphogenesis

**DOI:** 10.1371/journal.pgen.1006350

**Published:** 2016-10-03

**Authors:** Elizabeth J. Polvi, Anna F. Averette, Soo Chan Lee, Taeyup Kim, Yong-Sun Bahn, Amanda O. Veri, Nicole Robbins, Joseph Heitman, Leah E. Cowen

**Affiliations:** 1 Department of Molecular Genetics, University of Toronto, Toronto, Ontario, Canada; 2 Department of Molecular Genetics and Microbiology, Medicine, and Pharmacology and Cancer Biology, Duke University Medical Center, Durham, North Carolina, United States of America; 3 Department of Biology, University of Texas at San Antonio, San Antonio, Texas, United States of America; 4 Department of Biochemistry, College of Life Science and Biotechnology, Yonsei University, Seoul, Republic of Korea; Concordia University, CANADA

## Abstract

Fungal pathogens have evolved diverse strategies to sense host-relevant cues and coordinate cellular responses, which enable virulence and drug resistance. Defining circuitry controlling these traits opens new opportunities for chemical diversity in therapeutics, as the cognate inhibitors are rarely explored by conventional screening approaches. This has great potential to address the pressing need for new therapeutic strategies for invasive fungal infections, which have a staggering impact on human health. To explore this approach, we focused on a leading human fungal pathogen, *Candida albicans*, and screened 1,280 pharmacologically active compounds to identify those that potentiate the activity of echinocandins, which are front-line therapeutics that target fungal cell wall synthesis. We identified 19 compounds that enhance activity of the echinocandin caspofungin against an echinocandin-resistant clinical isolate, with the broad-spectrum chelator DTPA demonstrating the greatest synergistic activity. We found that DTPA increases susceptibility to echinocandins via chelation of magnesium. Whole genome sequencing of mutants resistant to the combination of DTPA and caspofungin identified mutations in the histidine kinase gene *NIK1* that confer resistance to the combination. Functional analyses demonstrated that DTPA activates the mitogen-activated protein kinase Hog1, and that *NIK1* mutations block Hog1 activation in response to both caspofungin and DTPA. The combination has therapeutic relevance as DTPA enhanced the efficacy of caspofungin in a mouse model of echinocandin-resistant candidiasis. We found that DTPA not only reduces drug resistance but also modulates morphogenesis, a key virulence trait that is normally regulated by environmental cues. DTPA induced filamentation via depletion of zinc, in a manner that is contingent upon Ras1-PKA signaling, as well as the transcription factors Brg1 and Rob1. Thus, we establish a new mechanism by which metal chelation modulates morphogenetic circuitry and echinocandin resistance, and illuminate a novel facet to metal homeostasis at the host-pathogen interface, with broad therapeutic potential.

## Author Summary

Invasive fungal infections pose a serious threat to human health worldwide, with *Candida albicans* being a leading fungal pathogen. Mortality is in part due to the limited arsenal of effective antifungals, with drug resistance on the rise. The echinocandins, which target the fungal cell wall, are the newest class of antifungal, and echinocandin resistance has already emerged. Here, we screened a library of 1,280 pharmacologically active compounds to identify those that potentiate echinocandin activity against an echinocandin-resistant isolate. The lead compound was a chelator, DTPA, which affects resistance by depleting magnesium. Genome sequencing of mutants resistant to the combination of DTPA and echinocandin revealed mutations in the gene encoding Nik1, which signals upstream of the Hog1 stress response pathway. We established that DTPA acts through Nik1 to modulate Hog1 signaling and enhance echinocandin activity, and that this combination has therapeutic benefits in a murine model of candidiasis. We also discovered that DTPA modulates *C*. *albicans* morphogenesis, a key virulence trait. DTPA induced filamentation by chelating zinc, in a manner that is contingent upon core filamentation pathways and specialized circuitry. Thus, we establish novel roles for metal homeostasis in *C*. *albicans* pathogenesis, thereby illuminating new therapeutic strategies for life-threatening infectious disease.

## Introduction

Invasive fungal infections have a devastating impact on human health worldwide. The most vulnerable individuals are those suffering from immune deficiencies due to chemotherapy for cancer, immunosuppression for transplants of solid organs or stem cells, or infection with HIV [1]. The incidence of deadly invasive fungal infections is on the rise, in concert with the increasing use of immunosuppressive measures and invasive medical procedures [2,3]. Immunocompetent individuals are also at risk, especially those in the expanding adult-onset diabetic population. Approximately 1.5 million people die every year from invasive fungal infections, which exceeds the death toll of malaria or tuberculosis [1]. *Candida* species are a leading cause of mycotic death worldwide, and account for over 85% of all hospital acquired fungal infections [2]. *Candida albicans* is the primary cause of systemic candidiasis with mortality rates of ~40% [4,5], even with current treatment options.

There is a limited repertoire of antifungal drugs available to treat human fungal infections, with the utility of current drugs restricted by problems of host toxicity, fungistatic activity, or drug resistance. There are only three major antifungal drug classes for treatment of invasive infections, with the development of novel classes of antifungals having largely stalled since the 1990s [6]. The polyenes were discovered more than 50 years ago, and have fungicidal activity due to binding and extracting ergosterol from fungal cell membranes, with host toxicity resulting from collateral effects on cholesterol in human cell membranes [7]. The first azoles were developed in the 1970s [8], and exert fungistatic activity by inhibiting the ergosterol biosynthetic enzyme lanosterol 14α-demethylase; they are the most widely deployed class of antifungal, but are vulnerable to drug resistance given their fungistatic activity against many fungal pathogens [9]. While newer generation azoles have been introduced into the clinic more recently, they remain vulnerable to cross-resistance across the azole class [10]. The echinocandins were first introduced into the clinic in the early 2000s, and impair fungal cell wall integrity by inhibiting biosynthesis of a structural polysaccharide, (1,3)-β-D-glucan [11]. Echinocandins remain a front-line therapy for invasive candidiasis, and thus the emergence of echinocandin resistance in *Candida* poses grave concern [12,13].

Echinocandin resistance is increasing in prevalence in the clinic. In *C*. *albicans*, resistance is often attributable to mutations in *FKS1*, which encodes the drug target (1,3)-β-D-glucan synthase [14–16]. Resistance phenotypes can also be modulated by compensatory changes in the fungal cell wall, as with the reduced echinocandin susceptibility that accompanies elevated chitin production [17]. Both the basal tolerance of wild-type strains to echinocandins, as well as the echinocandin resistance of *FKS1* mutants is contingent upon the capacity to sense and respond to drug-induced cell wall stress. Crucial cell wall stress responses include the protein kinase C (PKC) cell wall integrity cascade, the calcineurin signaling pathway, and the high osmolarity glycerol response (HOG) pathway, which converge on regulation of chitin synthesis in response to cell wall stress [18,19]. Signaling through these pathways is controlled by the molecular chaperone Hsp90, which is required for stability and activation of Hog1, calcineurin, and the terminal mitogen-activated protein kinase (MAPK) in the PKC signaling pathway, Mkc1 [20–22]. Perturbation of these stress response pathways causes hypersensitivity to the cell wall stress induced by echinocandins [12,20,23,24], suggesting that small molecules that inhibit key stress response regulators hold great potential as combination therapy agents to enhance antifungal efficacy and reverse drug resistance [25,26]. The therapeutic utility of current inhibitors of these stress response regulators as antifungals is compromised by lack of fungal selectivity, with host toxicity or immunosuppressive effects resulting from inhibition of the human counterparts. Thus, there is an urgent need for the discovery of new molecules that can increase the efficacy of echinocandins and overcome resistance.

The discovery of molecules that impair drug resistance or virulence traits generates new opportunities for chemical diversity in therapeutic agents, as they are not typically explored by conventional screening approaches for molecules that target essential processes [27,28]. Beyond expanding the repertoire of antifungal agents, targeting drug resistance regulators offers additional benefits such as minimizing effects on the host mycobiome and reducing selection pressure for the evolution of drug resistance. These same benefits apply to targeting virulence factors [29]. For *C*. *albicans*, a central virulence factor is the capacity to transition between yeast and filamentous growth [30,31], with most mutants that are locked in either morphology being attenuated in virulence [32–35]. Filamentation is induced by environmental cues such as serum, alkaline pH, and elevated CO_2_ in a manner that is contingent upon elevated temperature [9]. Hsp90 is a key regulator of temperature-dependent morphogenesis, with profound effects on core morphogenetic circuitry including the Ras1-protein kinase A (PKA) signaling cascade [36,37]. As has recently come to light through systematic screens of chemical combinations [28], the discovery of molecules that enhance the activity of existing antifungals and also modulate virulence traits offers great potential to bolster the antifungal pipeline.

In this study, we screened a library of 1,280 pharmacologically active compounds to identify those that increase the efficacy of caspofungin against an echinocandin-resistant *C*. *albicans* clinical isolate. We identified 19 compounds that potentiate the activity of caspofungin with negligible antifungal activity on their own. We focused our analysis on the broad-spectrum chelator DTPA, which had the greatest synergistic activity. Reducing levels of magnesium alone was sufficient to impair growth in the presence of caspofungin, suggesting that DTPA potentiates echinocandin activity via magnesium chelation. To identify effectors through which DTPA modulates echinocandin activity, we selected for spontaneous mutants resistant to the combination of DTPA and caspofungin. Whole genome sequencing coupled with functional analyses revealed that mutations in the histidine kinase gene *NIK1* confer resistance to the drug combination. While both caspofungin and DTPA activate the MAPK Hog1, *NIK1* mutations block activation in response to either compound, suggesting that DTPA enhances the efficacy of caspofungin by modulating Hog1 signaling through Nik1. We found that DTPA not only synergizes with caspofungin, but also induces filamentation in the absence of elevated temperature or other inducing cues. DTPA induced filamentation via depletion of zinc. To identify the circuitry through which DTPA induces filamention we performed a genetic screen of 143 homozygous deletion mutants, which revealed that DTPA-induced filamentation is contingent upon Ras1-PKA signaling, as well as the transcription factors Brg1 and Rob1. Together, our findings establish a new mechanism by which chelation of trace metals modulates fungal morphogenesis and cellular responses to drug-induced stress, and reveals a new way in which metal homeostasis impacts host-pathogen interactions.

## Results

### Screening a library of 1,280 pharmacologically active compounds identifies molecules that potentiate caspofungin efficacy against an echinocandin-resistant clinical isolate

**Fig 1:**
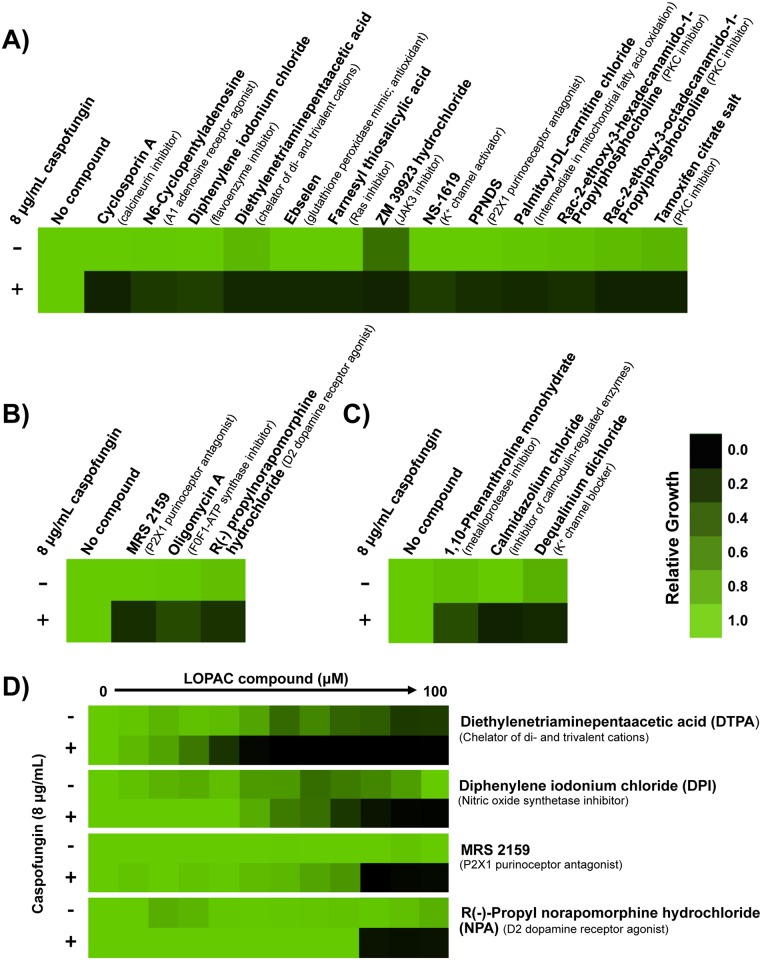
Screening a library of 1,280 pharmacologically active compounds identifies molecules that potentiate the activity of caspofungin against an echinocandin-resistant clinical isolate. **A)** The LOPAC^1280^ Navigator library was screened at 25 μM in RPMI medium in the presence or absence of 8 μg/mL caspofungin to identify compounds that enhance the efficacy of caspofungin against an echinocandin-resistant *C*. *albicans* clinical isolate (CaLC990). Growth was measured by absorbance at 600 nm after 96 hours at 30°C. A compound was classified as a hit if it inhibited growth by 90% in combination with caspofungin compared to growth with the LOPAC compound alone. Optical densities were normalized relative to the no compound control for wells with LOPAC compound alone, or to the caspofungin-only control for wells with caspofungin and LOPAC compound. Data was displayed as a heat map using Java TreeView 1.1.6, see color bar. **B)** Compounds identified as ‘weak hits’ (inhibited growth by 80–90% in combination with caspofungin compared to growth with the LOPAC compound alone) were rescreened at 50 μM of the LOPAC compound and analyzed as in A). **C)** Compounds that were toxic on their own (inhibited growth by greater than 70% in the absence of caspofungin) were rescreened at 12.5 μM and analyzed as in A). **D)** A dose response assay was used to quantify the impact of lead compounds on caspofungin resistance. Shown are the four compounds that best reduced the growth of the resistant strain in combination with caspofungin, and that have not previously been implicated in drug resistance. Assays were performed in RPMI medium in the presence or absence of a fixed concentration of caspofungin (8 μg/mL), as indicated. Data was analyzed after 96 hours at 30°C and normalized as in Part A.

We first aimed to identify compounds that enhance echinocandin activity against a *C*. *albicans* caspofungin-resistant clinical isolate. We used an isolate with an *FKS1*^*T1922C*^ homozygous mutation, resulting in the amino acid substitution of Fks1^F641S^, thus requiring a high concentration of caspofungin to achieve Fks1 inhibition [38]. We screened the LOPAC^1280^ Navigator Library. This was similar in approach to our previous screen to identify molecules that potentiate the activity of azoles, which revealed a new role for the PKC signaling pathway in enabling responses to cell membrane stress [21]. Similarly, our expectation was that a screen to identify compounds that potentiate caspofungin activity would reveal those that cripple stress response pathways crucial for responding to cell wall stress. We initially screened the library at 25 μM in RPMI medium at 30°C, in the presence or absence of 8 μg/mL caspofungin. This concentration of caspofungin inhibited the growth of the resistant clinical isolate by approximately 60%. This initial screen identified 13 compounds that were classified as strong hits based on two criteria: 1) they reduced growth in the presence of caspofungin by at least 90% compared to growth in the LOPAC compound alone; and 2) inhibited growth by less than 70% in the absence of caspofungin (Fig 1A). Strong hits included compounds that target key regulators of echinocandin resistance phenotypes, such as inhibitors of calcineurin and PKC signaling [19,20,23,24], validating our screen. The initial screen also identified nine compounds whose targets have not been previously implicated in echinocandin resistance phenotypes.

We performed additional analyses to increase our capacity to identify molecules that modulate echinocandin resistance phenotypes, and to validate lead compounds from the initial screen. For compounds from our initial screen that were classified as weak hits based on inhibition of growth by 80–90% in combination with caspofungin compared to growth with the LOPAC compound alone, we re-screened them at twice the concentration (50 μM); this identified three additional molecules that potentiate echinocandin antifungal activity (Fig 1B). For compounds from our initial screen that inhibited growth in the absence of echinocandin by greater than 70%, we re-screened them at half the concentration (12.5 μM); this identified another three compounds that enhance echinocandin antifungal activity (Fig 1C). Finally, to validate all of the lead compounds from the screen, we performed minimum inhibitory concentration (MIC) assays in the absence or presence of 8 μg/mL caspofungin to quantify the magnitude of impact on echinocandin resistance of the clinical isolate (S1 Fig). The four compounds that best potentiate caspofungin activity against the clinical isolate and whose roles in echinocandin susceptibility have not been previously described, are shown in Fig 1D. The greatest activity was observed with diethylenetriamine pentaacetic acid (DTPA), a chelator of di- and trivalent cations [39].

### DTPA potentiates echinocandin activity via depletion of magnesium

**Fig 2:**
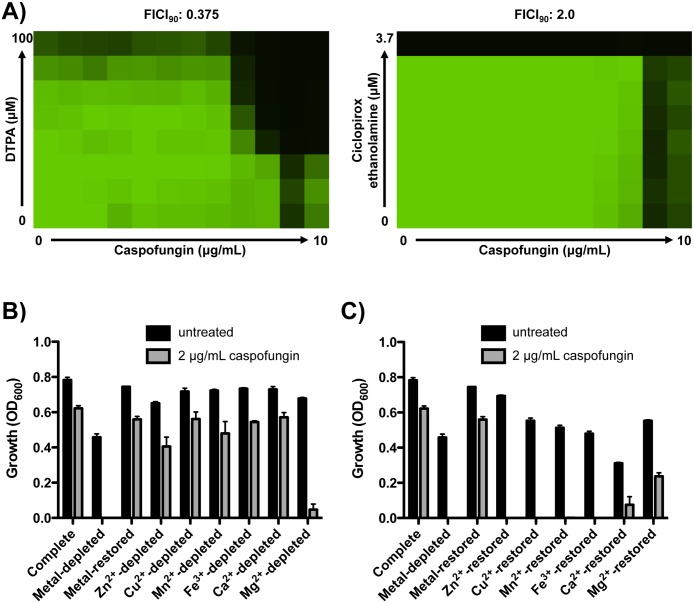
DTPA enhances caspofungin activity via depletion of magnesium. **A)** DTPA enhances the efficacy of caspofungin against an echinocandin-resistant clinical isolate, while ciclopirox ethanolamine does not. A dose response matrix (checkerboard assay) was performed in synthetic defined medium with gradients of caspofungin and DTPA or the iron chelator ciclopirox ethanolamine, as indicated. Data was analyzed after 72 hours at 30°C, and analyzed as in Fig 1A. **B)** Depleting magnesium impairs growth of a clinical isolate in caspofungin (P<0.001, two-way ANOVA, Bonferroni correction). Chelex 100 resin was used to deplete synthetic defined medium of its metal components. Metals were restored based on the concentrations at which they are normally present in yeast nitrogen base, restoring each individual metal as indicated. After 24 hours, optical density at 600 nm was assessed in each medium, in the absence or presence of 2 μg/mL caspofungin. Data are means ± SD for triplicate samples and are representative of two independent experiments. **C)** Addition of magnesium to metal-depleted medium best restores echinocandin resistance of the clinical isolate (P<0.001, two-way ANOVA, Bonferroni correction). Assay was performed and analyzed as in Fig 2B.

Our discovery of a broad-spectrum chelator as the strongest potentiator of echinocandin activity suggests that targeting metal homeostasis may provide a powerful therapeutic strategy. There is precedent for the use of chelators in the treatment of fungal infections, as with ciclopirox ethanolamine for treatment of superficial mycoses [40]. The broad-spectrum antifungal activity of ciclopirox ethanolamine is thought to be due to chelation of iron [41]. To determine if the capacity to enhance echinocandin activity is a general property of chelators we performed dose response matrices (or checkerboard assays) with DTPA or ciclopirox ethanolamine and caspofungin against the echinocandin-resistant *C*. *albicans* clinical isolate. A fractional inhibitory concentration index (FICI) is an expression of the combinatorial effect of two compounds. A value ≤0.5 indicates synergy, while a value >0.5–4.0 indicates indifference [42]. DTPA and caspofungin were synergistic (FICI = 0.375), while ciclopirox ethanolamine had no impact on caspofungin resistance (FICI = 2.0) (Fig 2A). This suggests that DTPA has a distinct mode of action from other chelators in clinical use as antifungals, and that DTPA modulates echinocandin resistance phenotypes via chelation of metal cations distinct from iron.

To determine the identity of the metal cation(s) through which DTPA modulates echinocandin resistance, we used an ion exchange resin (Chelex 100 resin) to deplete synthetic defined medium of its metal components. We then selectively restored metals based on the concentrations at which they are normally present in the medium, and assessed the growth of the echinocandin-resistant clinical isolate in the presence or absence of 2 μg/mL caspofungin (Fig 2B). In medium containing all metal components (Complete), this concentration of caspofungin did not inhibit growth. Depletion of all metals completely blocked growth in the presence of caspofungin; importantly, robust growth was observed in the absence of caspofungin, indicating that sufficient trace metals remain in the metal-depleted medium to support growth. Addition of all metals to the metal-depleted medium restored growth in the presence of caspofungin. Depleting each metal individually (creating ‘drop out’ media by addition of metals to the metal-depleted medium) revealed that depletion of magnesium is sufficient to impair growth of the echinocandin resistant clinical isolate in the presence of caspofungin (P <0.001, two-way ANOVA, Bonferroni correction) (Fig 2B). Conversely, addition of each metal individually to the metal-depleted medium revealed that magnesium best restores echinocandin resistance of the clinical isolate (P <0.001, two-way ANOVA, Bonferroni correction) (Fig 2C). We performed a comparable analysis with a wild-type strain of *C*. *albicans*, and confirmed that addition of magnesium to metal-depleted medium best restores growth in caspofungin (P <0.001, two-way ANOVA, Bonferroni correction) (S2 Fig). These results suggest that DTPA potentiates echinocandin antifungal activity primarily through the depletion of magnesium, and demonstrate that modulation of metal availability provides a powerful strategy to enhance antifungal activity. Because the efficacy of chelators such as DTPA does not require intracellular accumulation, this approach will evade efflux-mediated resistance.

### Genome sequencing of mutants resistant to the combination of caspofungin and DTPA identifies mutations in *YKE2* and *NIK1*

**Fig 3:**
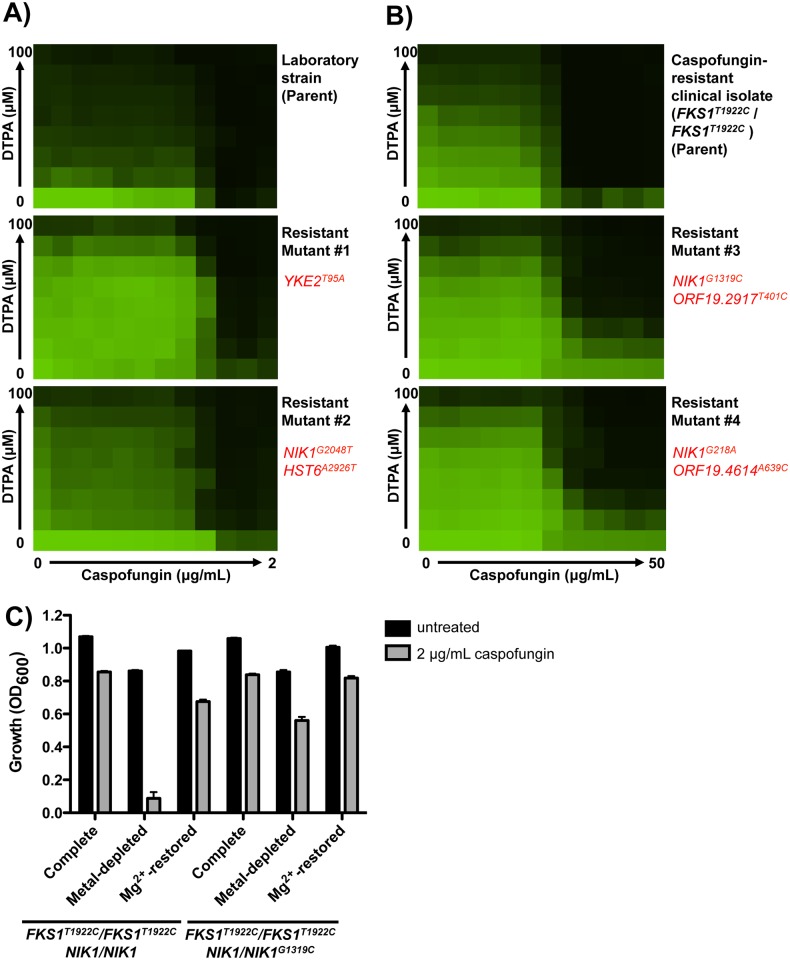
Whole-genome sequencing identifies mutations that confer resistance to DTPA and caspofungin. **A)** Mutants resistant to the combination of DTPA and caspofungin in a wild-type strain background (SN95) were obtained by plating 2x10^8^ cells on RPMI plates with a concentration of 100 μM DTPA and 0.25 μg/mL caspofungin. Spontaneous mutants were selected after 4 days at 30°C. The drug susceptibility profiles of the resistant mutants were verified by dose response matrix (checkerboard) analysis. Cells were grown in RPMI at 30°C. Optical densities (OD_600_) were measured after 72 hours and normalized to the no drug control. Data was displayed as a heat map, as in Fig 1. Whole genome sequencing identified heterozygous mutations in the mutant strains relative to the parental strains. Non-synonymous mutations located within open reading frames are indicated in red. **B)** Mutants resistant to the combination of DTPA and caspofungin in a caspofungin-resistant clinical isolate background (*FKS1*^*T1922C*^*/FKS1*^*T1922C*^) were obtained following the same basic protocol as in A), but on RPMI plates with 100 μM DTPA and 8 μg/mL caspofungin. Checkerboard analysis was performed as in A). **C)** The *NIK1/NIK1*^*G1319C*^ mutation in the *FKS1*^*T1922C*^*/FKS1*^*T1922C*^ background confers resistance to the combination of metal-depletion and caspofungin. (P<0.001, two-way ANOVA, Bonferroni correction). Chelex 100 resin was used to deplete synthetic defined medium of its metal components, as in Fig 2B. Cells were grown in RPMI at 30°C for 72 hours.

As an unbiased approach to further investigate the mechanism by which DTPA exerts synergistic activity with caspofungin, we selected for mutants resistant to the combination. We plated 2x10^8^ cells of a standard laboratory strain and the caspofungin-resistant clinical isolate on medium containing a high concentration of DTPA (100 μM) and caspofungin (0.25 μg/mL for the laboratory strain and 8 μg/mL for the resistant isolate). We quantified resistance to DTPA, caspofungin, and the combination using dose response matrices (checkerboards). We recovered two mutants in the laboratory strain background, which had increased resistance to both DTPA and caspofungin individually, as well as in combination (Fig 3A). We also recovered two mutants in the echinocandin-resistant clinical isolate background, which both had increased resistance to DTPA alone and in combination with caspofungin (Fig 3B).

To identify mutations that confer resistance to the combination of DTPA and caspofungin, we performed whole genome sequencing on the two mutants from each background as well as on the two parental strains. Sequence reads were aligned to the published *C*. *albicans* genome (SC5314, assembly 21, with the mean depth of coverage being 106X for all assembled sequences). We identified only one or two non-synonymous single nucleotide variants within coding regions in the mutants relative to their parents (Fig 3A and 3B). All of these mutations were heterozygous. Strikingly, three of the four sequenced mutants harbored mutations in the *NIK1* gene, which encodes one of three *C*. *albicans* histidine kinases involved in the two-component phosphorelay system that regulates the Hog1 MAPK stress response pathway [43–46]. Two of the identified *NIK1* mutations result in substitutions in the histidine kinase HAMP linker domain (G73D and R440T), and the third Nik1 substitution is found within the ATP-binding domain (G683V). The HAMP domains of CaNik1 have been found previously to mediate sensitivity to various fungicides [47]. The fourth mutant harbored a mutation in *YKE2*, which encodes a component of the prefoldin complex in *Saccharomyces cerevisiae* [48,49].

Next, we performed allele replacements to functionally validate the mutations identified by whole genome sequencing of the mutants in the laboratory strain background. We replaced one native allele of *NIK1* or *YKE2* in the parental strain with the corresponding allele from the resistant mutants. Both the *YKE2*^*T95A*^ and *NIK1*^*G2048T*^ alleles conferred resistance to DTPA and caspofungin in the laboratory strain background (S3A Fig). Because the phenotypic effects were observed in heterozygous mutants, the mutations likely do not cause loss of function. Consistent with this hypothesis, deletion of *NIK1* did not confer resistance to either DTPA or caspofungin (S3B Fig).

Finally, we assessed resistance of one of the *NIK1* point mutants to the combination of metal depletion and caspofungin. While the echinocandin-resistant clinical isolate (*FKS1*^*T1922C*^/*FKS1*^*T1922C*^) is unable to grow with the combination of metal depletion and caspofungin (Figs 2B and 3C), a heterozygous *NIK1*^*G1319C*^ mutation (resulting in the amino acid substitution Nik1^R440T^) restores this ability (P <0.001, two-way ANOVA, Bonferroni correction) (Fig 3C). Addition of magnesium also restores the ability of either strain to grow in the presence of caspofungin. Thus, specific mutations in *NIK1* block the effects of metal depletion on echinocandin resistance.

### *NIK1* mutations phenocopy impaired signaling through the Hog1 pathway

**Fig 4:**
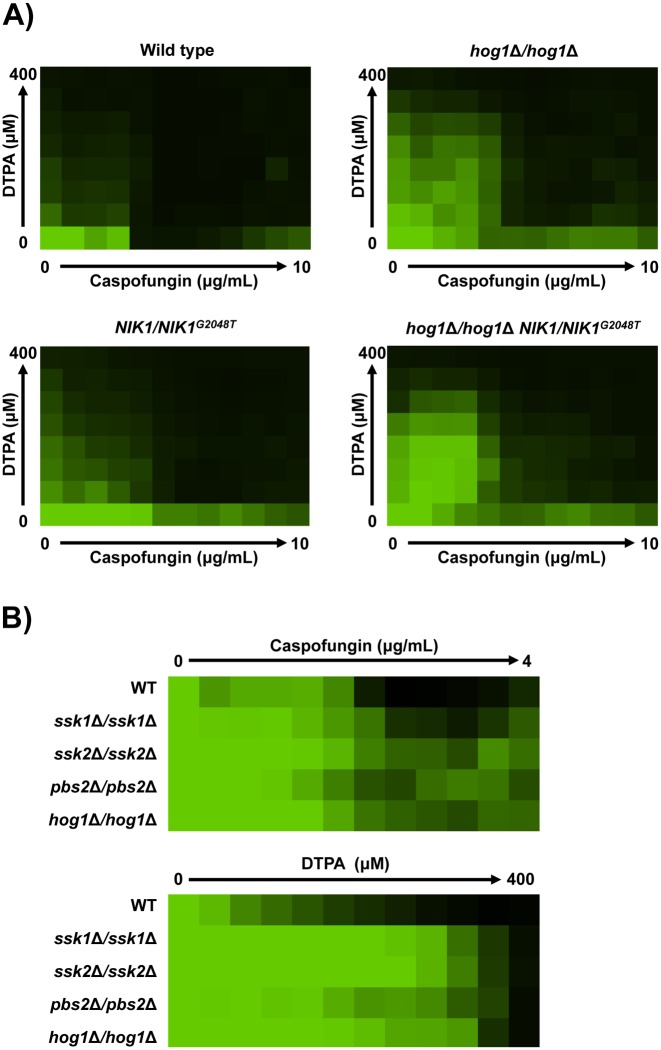
The *NIK1*^*G2048T*^ allele phenocopies deletion of *HOG1* or other components of the Hog1 signaling pathway. **A)** Resistance phenotypes (wild type SN250) were assessed by checkerboard analysis in RPMI medium containing gradients of DTPA and caspofungin concentrations. Data was analyzed after 48 hours at 30°C and normalized to the no drug control. Data was displayed as a heat map, as in Fig 1. **B)** Susceptibility of each strain (wild type SN250) to either caspofungin or DTPA was assessed over a gradient of concentrations in an MIC assay. Assays were performed in RPMI medium, and data was analyzed after 72 hours at 30°C and normalized as in Fig 1.

Nik1 is one of three *C*. *albicans* histidine kinases, along with Chk1 and Sln1 [45,46]. In *S*. *cerevisiae*, the sole histidine kinase Sln1 is the osmosensor that regulates the Hog1 MAP kinase cascade [50,51]. In *C*. *albicans*, the histidine kinases are also thought to signal upstream of Hog1, although the exact role for Nik1 remains unknown as does the identity of the osmosensor (reviewed in [46] and [52]). Deletion of *HOG1* in *C*. *albicans* confers resistance to cell wall stressors such as Calcofluor White and Congo Red [53–55], suggesting that the *NIK1* point mutations may modulate resistance via Hog1. To test this, we assayed resistance of a *hog1Δ/hog1Δ* homozygous deletion mutant to both caspofungin and DTPA. We found that the *hog1Δ/hog1Δ* mutant had increased resistance to the combination of DTPA and caspofungin compared to its parental strain, as does the *NIK1/NIK1*^*G2048T*^ mutant (Fig 4A). Furthermore, deletion of other components of the Hog1 signaling pathway such as the genes encoding Ssk1, Ssk2, or Pbs2 had comparable effects on resistance to DTPA and caspofungin as did deletion of Hog1 (Fig 4B). This suggests that the *NIK1*^*G2048T*^ allele may confer resistance via effects on Hog1. Consistent with this model, introducing the *NIK1*^*G2048T*^ allele into the *hog1Δ/hog1Δ* mutant did not further increase resistance to either DTPA or caspofungin (Fig 4A). The finding that point mutations in *NIK1* phenocopy impaired signaling through the Hog1 pathway suggests that these mutations may confer resistance to DTPA and caspofungin by blocking Hog1 activation.

### *NIK1* mutations confer resistance to the combination of DTPA and caspofungin by blocking Hog1 activation in response to these compounds

**Fig 5:**
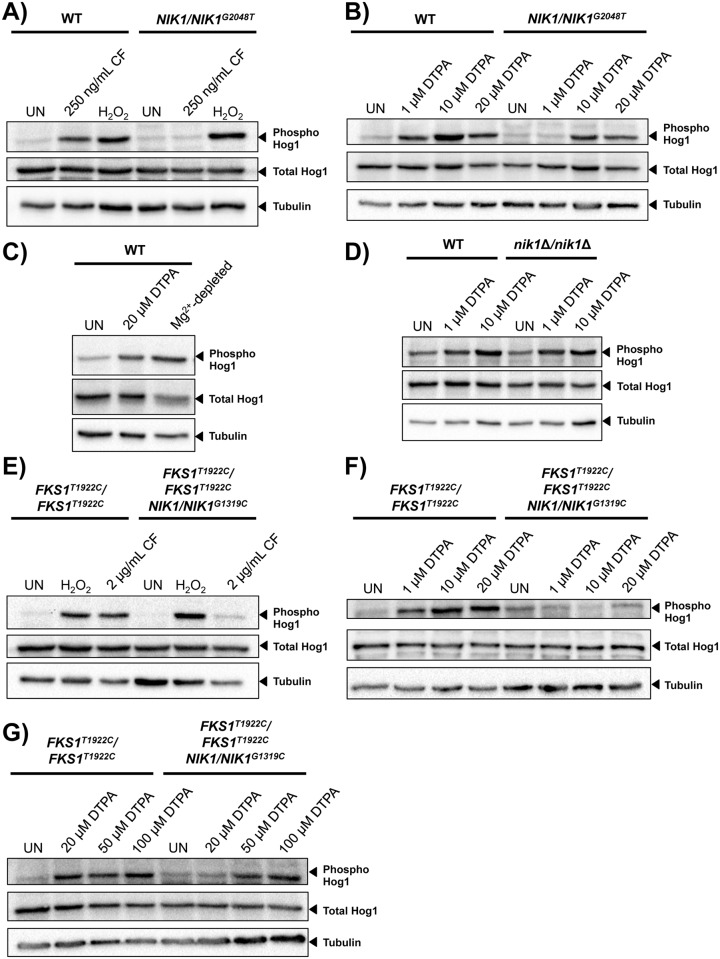
*NIK1* point mutations impair Hog1 activation in response to DTPA and caspofungin. **A)** Hog1 is phosphorylated in the wild type (SN95) in response to caspofungin (CF, 250 ng/mL), but not in the *NIK1/NIK1* ^*G2048T*^ mutant. (UN; untreated). Hog1 is phosphorylated in both the wild type and the mutant in response to H_2_O_2_ (10 mM). **B)** The *NIK1*^*G2048T*^ allele impairs Hog1 activation in response to 1 μM DTPA, but not higher concentrations of DTPA. **C)** Depletion of magnesium is sufficient to induce Hog1 phosphorylation. Wild-type cells were grown in SD medium in the presence of 20 μM DTPA, or in Chelex-treated medium with all metals restored except magnesium. **D)** Deletion of *NIK1* does not impair Hog1 phosphorylation in response to DTPA. **E)** The *NIK1*^*G1319C*^ allele impairs Hog1 activation in response to 2 μg/mL caspofungin in the caspofungin-resistant clinical isolate (*FKS1*^*T1922C*^*/FKS1*^*T1922C*^). Hog1 is phosphorylated in both the wild type and the mutant in response to H_2_O_2_ (10 mM). **F)** The *NIK1*^*G1319C*^ allele impairs Hog1 activation in response to DTPA concentrations up to 20 μM. **G)** High concentrations of DTPA (50 μM and 100 μM) cause Hog1 activation in the *NIK1/NIK1*^*G1319C*^ mutant. For all blots, total protein was extracted and resolved by SDS-PAGE, and blots were hybridized with α-phospho-p38 MAPK to monitor phosphorylated Hog1, α-Hog1 to monitor total Hog1, or α-tubulin as a loading control. In all lanes, 40 μg of protein was loaded, except for C) where 15 μg was loaded.

To determine if the *NIK1*^*G2048T*^ mutation impairs Hog1 function, we first monitored phosphorylation of Hog1 in response to both caspofungin and the oxidative stress-inducing agent hydrogen peroxide in the laboratory strain. Hog1 is known to be activated in response to both hydrogen peroxide and caspofungin [56,57]. While both stressors cause an increase in Hog1 phosphorylation in the wild-type strain, only hydrogen peroxide induced Hog1 phosphorylation in the *NIK1/NIK1*^*G2048T*^ mutant (Fig 5A). This suggests that the *NIK1* mutation blocks signaling of caspofungin-induced stress through the Hog1 pathway. Next, given that deletion of *HOG1* confers resistance to DTPA, we tested whether DTPA activates Hog1. All concentrations of DTPA tested induced Hog1 phosphorylation in the wild-type strain, but this activation was impaired in the *NIK1* ^*G2048T*^ mutant, such that a higher concentration of DTPA was required to achieve activation (Fig 5B). Notably, the ability of DTPA to activate Hog1 was phenocopied by depletion of magnesium (Fig 5C), providing additional evidence that DTPA affects Hog1 signaling via chelation of magnesium. Further, phosphorylation of Hog1 in response to DTPA was maintained in a *nik1*Δ*/nik1*Δ homozygous deletion mutant indicating that G2048T is not a *NIK1* loss-of-function mutation (Fig 5D). Thus, the G2048T *NIK1* mutation impairs activation of Hog1 in response to specific cellular stressors.

We also monitored Hog1 activation in the echinocandin-resistant isolate (*FKS1*^*T1922C*^*/ FKS1*^*T1922C*^). Although 2 μg/mL caspofungin activated Hog1 in this parental background, activation of Hog1 was blocked in the *NIK1*^*G1319C*^ mutant that is resistant to the combination of DTPA and caspofungin (Fig 5E). As with the laboratory strain, the *NIK1* mutation did not alter activation of Hog1 in response to hydrogen peroxide, but did impair Hog1 activation in response to DTPA in the echinocandin-resistant isolate. Concentrations of DTPA up to 20 μM resulted in Hog1 activation in the parent, but not in the *NIK1*^*G1319C*^ mutant (Fig 5F); Hog1 remains capable of activation in response to higher concentrations of DTPA in the *N1K1* mutant (Fig 5G). The *NIK1* alleles in the wild-type and *FKS1*^*T1922C*^*/ FKS1*^*T1922C*^ backgrounds are different, and thus may have distinct magnitudes of effect on Hog1 activation in response to DTPA. Together, these results demonstrate that both DTPA and caspofungin activate Hog1, and that mutations in *NIK1* impair this activation in response to specific cues.

Given that metal cations such as magnesium are known to bind to cell wall components [58], we the explored the possibility that DTPA might non-specifically modulate other stress response pathways or act extracellularly on the cell wall, thereby potentiating caspofungin activity. We first assessed whether DTPA impairs signaling through the PKC cell wall integrity pathway. In *S*. *cerevisiae*, exposure to caspofungin induces phosphorylation of the MAPK Slt2 [59]. We monitored phosphorylation of the *C*. *albicans* ortholog, Mkc1, by Western blot. As expected, Mkc1 was phosphorylated in response to caspofungin (S4A Fig); DTPA did not induce activation of Mkc1 nor did it block activation in response to caspofungin. Additionally, we examined the effect of DTPA on cell wall architecture. Under conditions of cell wall stress, such as exposure to cell surface-perturbing agents, the levels of chitin and glucan in the cell wall increase dramatically [60]. We monitored cell wall chitin and glucan levels in the caspofungin-resistant isolate that was grown in RPMI and treated overnight with 50 μM DTPA or 0.32 μg/mL of caspofungin. Cells were stained with Aniline Blue to measure levels of exposed glucans, with Calcofluor White to measure chitin levels, or with Concanavalin A to measure levels of mannans. While treatment with the cell wall-targeting drug caspofungin drastically increased the levels of exposed glucan and chitin, treatment with DTPA had minimal effect (S4B Fig). Thus, DTPA activates Hog1 signaling but does not promiscuously activate the cell wall integrity pathway or cell wall remodelling.

### DTPA induces *C*. *albicans* filamentation via depletion of zinc

**Fig 6:**
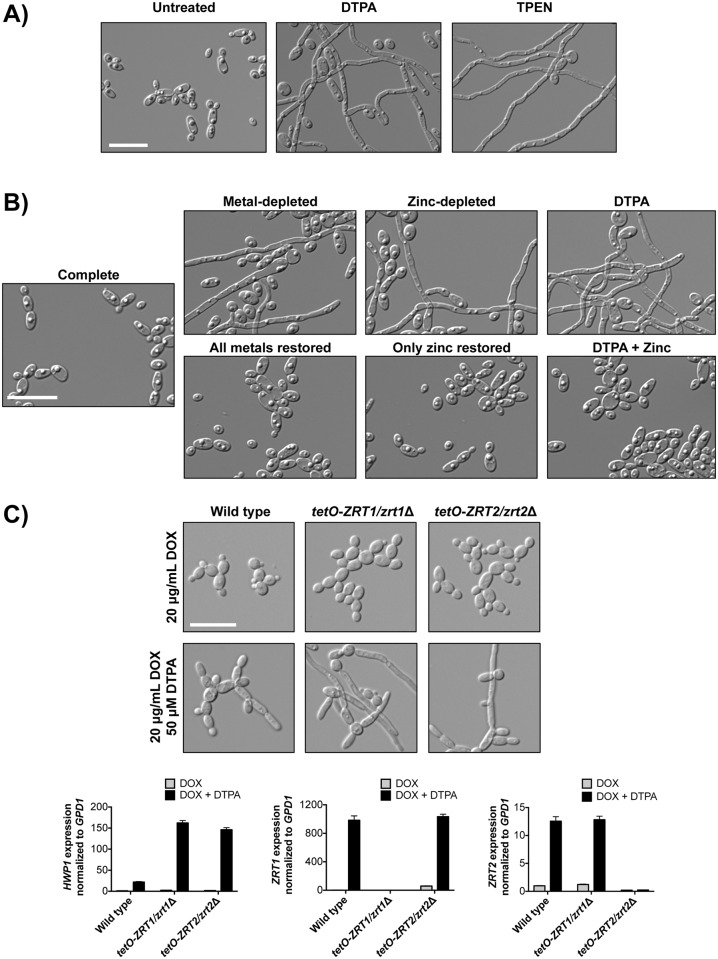
Depletion of zinc induces filamentous growth of *C*. *albicans*. **A)** DTPA and the zinc chelator TPEN induce filamentation. Wild-type cells (SN95) were grown in synthetic defined medium with 100 μM DTPA or 10 μM TPEN at 30°C for 24 hours. **B)** Zinc depletion induces filamentation. Wild-type cells were grown in untreated synthetic defined medium (Complete) or that treated with Chelex 100 resin to deplete metals (Metal-depleted). All metals, all metals except for zinc, or only zinc were restored, as indicated. DTPA-treated cells were grown in 50 μM DTPA, in the presence or absence of 8.7 mg/L ZnSO_4_. Cells were grown for 24 hours at 30°C. **C)** Transcriptional repression of the zinc transporters *ZRT1* or *ZRT2* renders cells hyperfilamentous in DTPA. Strains in which the only allele of *ZRT1* or *ZRT2* is under the control of a tetracycline-repressible promoter, and their wild-type counterpart (CaSS1), were grown for 24 hours in YPD at 30°C in the presence of 20 μg/mL doxycycline (DOX) and the presence or absence of 10 μM DTPA, and subcultured into YPD with 20 μg/mL DOX, in the presence or absence of 50 μM DTPA for 4.5 hours at 30°C. In both the *ZRT1* and *ZRT2* depletion strains, *HWP1* expression is increased in the presence of DOX and DTPA, relative to the wild type (P<0.0001, two-way ANOVA, Bonferroni correction). All scale bars are 20 μM.

As DTPA has profound effects on *C*. *albicans* drug resistance, we next assessed whether it also impacts another key *C*. *albicans* virulence trait: the capacity to transition between yeast and filamentous growth. We found that DTPA induced robust filamentation in wild-type cells (Fig 6A). Strikingly, this yeast-to-filament transition occurs even at 30°C and does not require a concurrent shift to 37°C, as do most other filament-inducing cues. In order to identify the metal through which DTPA induces filamentation, we used the Chelex 100 ion exchange resin to deplete synthetic defined medium of its metal components. As expected, the cells grew in the yeast form in complete medium; depletion of all metals induced filamentous growth, and addition of all metal components back to the metal-depleted medium restored growth in the yeast form (Fig 6B and S5 Fig). By omitting each metal individually from the metal supplement, we found that zinc-depletion induced robust filamentous growth. Conversely, addition of only zinc to metal-depleted medium repressed filamentation (Fig 6B and S5 Fig). Similarly, addition of excess zinc to DTPA-treated cells inhibited filamentation in response to DTPA (Fig 6B and S5 Fig). We used a lower concentration of DTPA (50 μM) that still induced filamentous growth, while minimizing the amount of metal and chelator present in the culture, making the add-back feasible. Depleting the medium of the other metals individually had minimal to no effect on cellular morphology, and only depletion of all metals together or depletion of zinc alone significantly increased filamentation compared to complete medium (S6 Fig) (P<0.0001, one-way ANOVA, Dunnett’s test). We further validated that depletion of zinc induces filamentous growth in two ways. First, we found that the zinc chelator TPEN induced filamentation (Fig 6A). Second, we demonstrated that doxycycline-mediated transcriptional repression of either of two zinc transporters, *ZRT1* and *ZRT2* [61–63], conferred hypersensitivity to the filament-inducing effects of DTPA (Fig 6C). We used a low concentration of DTPA (50 μM) to provide a mild filament-inducing cue in YPD in order to enable the identification of a hyperfilamentatous phenotype. We quantified this effect by measuring the expression of *HWP1*, which encodes hyphal cell wall protein 1, by quantitative RT-PCR. Upon treatment with DOX and DTPA, *HWP1* expression was increased in the *ZRT1* and *ZRT2* depletion strains compared to the wild type (P<0.0001, two-way ANOVA, Bonferroni correction). Treatment with DOX and DTPA also causes induction of *ZRT1* in the *ZRT2* depletion strain, and induction of *ZRT2* in the *ZRT1* depletion strain (P<0.0001, two-way ANOVA, Bonferroni correction), consistent with a transcriptional response to metal deficiency. Thus, depletion of zinc in the environment or impairment of zinc import induces *C*. *albicans* morphogenesis.

### Filamentation induced by DTPA is contingent upon the transcription factors Brg1, Rob1, and Efg1

**Fig 7:**
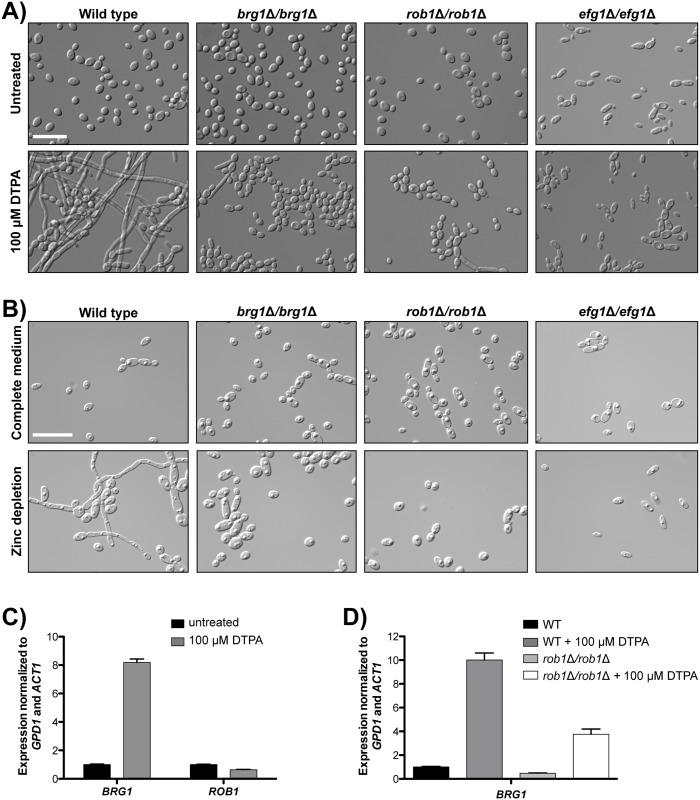
The transcription factors Brg1, Rob1 and Efg1 are required for DTPA-induced filamentation. **A)** A screen of 143 homozygous deletion mutants of transcription factor genes identified *brg1*Δ*/brg1*Δ, *rob1*Δ*/rob1*Δ, and *efg1*Δ*/efg1*Δ mutants as defective in filamentation in response to 100 μM DTPA in YPD at 30°C. Images were taken after 24 hours. Scale bar is 20 μM. **B)** The *brg1*Δ*/brg1*Δ, *rob1*Δ*/rob1*Δ, and *efg1*Δ*/efg1*Δ mutants are defective in filamentation in response to zinc depletion. Scale bar is 20 μM. **C)** *BRG1* transcript levels increase in response to DTPA, while *ROB1* levels do not (P <0.0001, unpaired t-test). Transcript levels of *BRG1* and *ROB1* were monitored by quantitative RT-PCR. Wild-type cells (SN95) were grown in YPD with or without 100 μM DTPA for 24 hours at 30°C, at which point they were subcultured into YPD with or without 100 μM DTPA for 4 hours. Transcript levels were normalized to *ACT1* and *GPD1*. Levels are expressed relative to the untreated samples, which are set to 1. Data are the means ± SD for triplicate samples and are representative of two independent experiments. **D)** Increased *BRG1* expression in DTPA-induced filaments is partially dependent upon Rob1 (P <0.001, one-way ANOVA, Bonferroni correction). *BRG1* transcript levels in the wild type (CaLC2740) and *rob1*Δ*/rob1*Δ mutants were analyzed as in B).

Morphogenesis in response to different cues is controlled by distinct cellular circuitry. To elucidate the circuitry required for DTPA-induced filamentation, we screened a library of 143 *C*. *albicans* homozygous deletion mutants of transcription factor genes [64]. Cells were grown in rich medium in static conditions at 30°C in the absence or presence of 50 μM DTPA, and morphology was assessed by microscopy. We identified three mutants that were defective in filamentation in response to DTPA: *brg1*Δ*/brg1*Δ, *rob1*Δ*/rob1*Δ, and *efg1*Δ*/efg1*Δ (Fig 7A). We also confirmed that these mutants are unable to filament in response to zinc depletion (Fig 7B). These three transcription factors are key components of a network that regulates the formation of biofilms [65], which are complex communities composed of multiple cellular morphologies that form upon adherence to surfaces [66]. Although Efg1 is a master regulator of morphogenesis and required for filamentation in response to most filament-inducing cues [67], Brg1 and Rob1 have more specialized roles in morphogenesis. For example, although *brg1*Δ*/brg1*Δ and *rob1*Δ*/rob1*Δ mutants are defective in biofilm formation, they are capable of filamentous growth in other contexts [65]. We confirmed that the *brg1*Δ*/brg1*Δ and *rob1*Δ*/rob1*Δ mutants filament in response to diverse cues (S7 Fig), suggesting that there is specificity to the role of Brg1 and Rob1 in morphogenesis induced by DTPA and zinc depletion.

To further delineate functional relationships between Brg1 and Rob1, we monitored *BRG1* and *ROB1* transcript levels in response to DTPA using qRT-PCR. We found that *BRG1* expression is induced in response to DTPA (P <0.0001, unpaired t-test), while *ROB1* expression is not (Fig 7C). Under biofilm conditions, Rob1 is required for expression of *BRG1* [65]. To determine if Rob1 also regulates *BRG1* expression in response to DTPA, we examined *BRG1* expression in the *rob1*Δ*/rob1*Δ mutants. Indeed, deletion of *ROB1* blocked induction of *BRG1* in response to DTPA (P <0.001, one-way ANOVA, Bonferroni correction) (Fig 7D). This reinforces the functional relationship between Rob1 and Brg1, and illuminates a new role for these regulators in morphogenesis in response to perturbation of metal homeostasis.

### DTPA-induced filamentation is associated with decreased Nrg1 levels and requires pathways involved in Nrg1 downregulation and degradation

**Fig 8:**
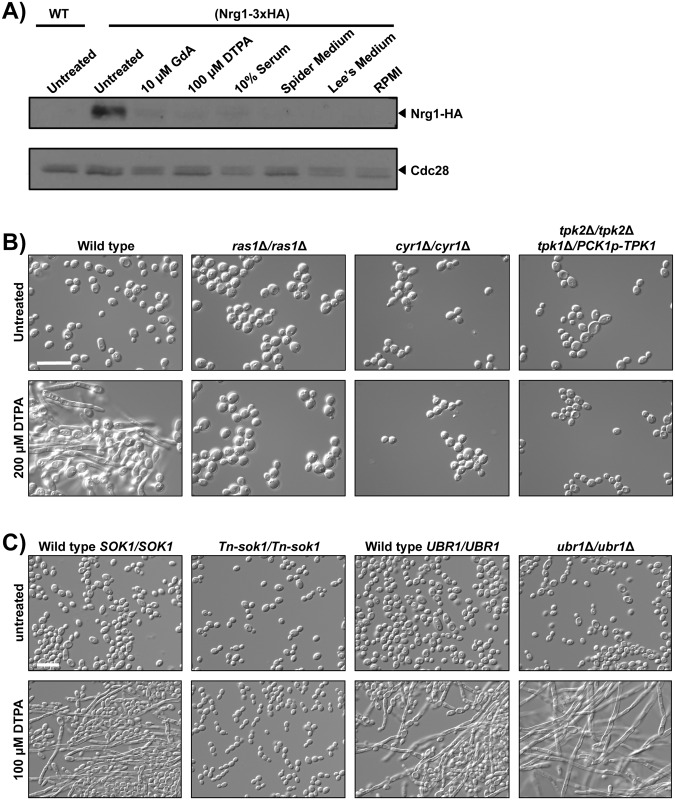
Filamentation induced by DTPA is associated with a decrease in Nrg1 protein level, and requires the cAMP-PKA pathway and the kinase Sok1. **A)** Nrg1 levels are reduced in response to all filament-inducing cues tested, including DTPA. One allele of *NRG1* was C-terminally 3xHA tagged in the BWP17 background to monitor Nrg1 levels. Cells were grown overnight in YPD at 30°C (with 80 mg/L uridine) and were subcultured into the same medium, supplemented with 10 μM geldanamycin or with 100 μM DTPA, as indicated, for 24 hours. Geldanamycin-treated and DTPA-treated cultures were then subcultured in the same conditions at 30°C and grown for 4.5 hours. Untreated cells were left untreated, or were grown in YPD with 10% serum at 37°C, Spider Medium at 37°C, Lee’s Medium at 37°C or RPMI medium at 37°C, all for 4.5 hours. Western blot analysis was performed as in Fig 5, using α-HA to monitor total Nrg1 and α-PSTAIRE (Cdc28) as a loading control. In all lanes, 50 μg of protein was loaded. **B)** The cAMP-PKA pathway is required for filamentation induced by DTPA. Wild-type cells (CAI4) or mutants were grown in YPD with uridine in the presence or absence of 200 μM DTPA at 30°C for 24 hours. We find the CAI4 background to require a slightly higher concentration of DTPA (200 μM DTPA) to induce filamentation. The *PCK1* promoter is repressed in the presence of glucose. **C)** Sok1 is required for DTPA-induced filamentation while Ubr1 is not. The *Tn*-*sok1* transposon mutant and its wild-type counterpart (DAY286), and the *ubr1*Δ*/ubr1*Δ mutant and its wild-type counterpart (SN250), were initially grown in YPD with or without 100 μM DTPA at 30°C for 24 hours and subsequently subcultured into the same conditions for 24 hours. All scale bars are 20 μM.

A molecular feature of filaments induced by diverse cues is reduction in the levels of the transcriptional repressor Nrg1 [34,68,69]. Removal of Nrg1 from the promoters of filament-specific genes is thought to be required for initiation and maintenance of filamentous growth [70]. *BRG1* is one of the many genes repressed by Nrg1 [71]. To determine if Nrg1 degradation is also associated with DTPA-induced filaments, we used Western blot analysis to monitor levels of epitope tagged Nrg1 in cells exposed to diverse filament-inducing cues. We found that Nrg1 levels were reduced in cells exposed to each of the filament-inducing cues tested, including DTPA (Fig 8A).

Next we assessed whether filamentation in response to DTPA was contingent upon circuitry controlling Nrg1 levels. Nrg1 is regulated at both the transcriptional and protein level in filament-inducing conditions [69]. Upon initiation of filamentation at 37°C, *NRG1* transcription is downregulated by the Ras1-PKA pathway [70,72]. Components of this pathway include the adenylyl cyclase Cyr1, which synthesizes cAMP and is activated by Ras1, and the PKA complex, which consists of two catalytic subunits, Tpk1 and Tpk2 (reviewed in [9]). We assessed morphology of mutants lacking components of the Ras1-PKA pathway in response to DTPA. We found that homozygous deletion of *RAS1* or *CYR1*, or depletion of *TPK1* in a strain lacking *TPK2* blocked filamentation in response to DTPA (Fig 8B). This assay utilizes 200 μM DTPA instead of 100 μM, in order to induce filamentation in the CAI4 background. Thus, Ras1-PKA signaling is required for filamentation in response to DTPA, consistent with the central role of this cascade in morphogenesis in response to diverse cues [67].

**Fig 9:**
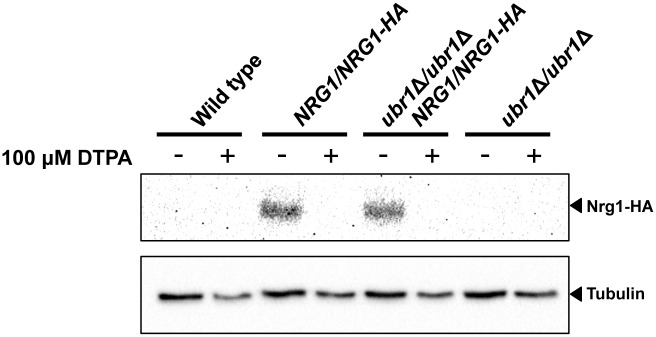
Nrg1 protein levels are reduced in DTPA-induced filamentous cells even in the absence of Ubr1. Wild-type (SN250) or *ubr1*Δ*/ubr1*Δ cells with one allele of HA-tagged Nrg1 were inoculated into YPD medium at 30°C for 4.5 hours in the presence or absence of 100 μM DTPA. Western blot analysis was performed as in Fig 5, using α-HA to monitor total Nrg1 and α-tubulin as a loading control. In all lanes, 40 μg of protein was loaded.

There is additional regulatory complexity to achieve rapid loss of Nrg1 upon the initiation of filamentation. The induction of filamentation at 37°C is accompanied by degradation of Nrg1 [70,72], which is controlled by a pathway that consists of the E3 ubiquitin ligase Ubr1, the transcriptional repressor Cup9, and the kinase Sok1 [72]. Ubr1 mediates degradation of Cup9, thereby alleviating transcriptional repression of *SOK1* and enabling Nrg1 degradation upon the initiation of filamentation [72]. To determine whether this pathway is required for filamentation induced by DTPA, as is the case with filamentation at 37°C, we monitored morphology of mutants of the positive regulators Ubr1 and Sok1. We found that *Tn*-*sok1/Tn-sok1* transposon insertion mutants were unable to filament in response to DTPA, however, the *ubr1Δ/ubr1Δ* homozygous deletion mutants filamented robustly (Fig 8C and S8 Fig). Nrg1 protein levels decreased in response to filamentation induced by DTPA, even in the absence of Ubr1 (Fig 9). This indicates that Ubr1 is dispensable for Nrg1 degradation in response to DTPA, and suggests that there is functional divergence in the regulation of Nrg1 degradation in response to distinct cues.

### DTPA enhances the efficacy of caspofungin in a murine model of caspofungin-resistant disseminated candidiasis

**Fig 10:**
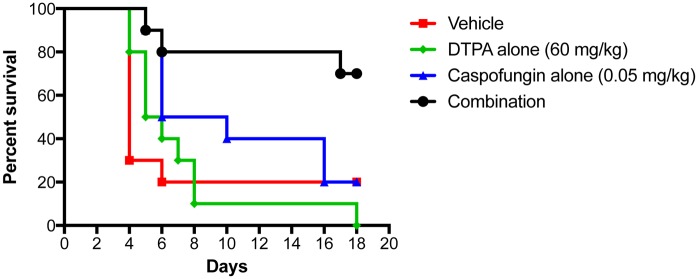
DTPA enhances the efficacy of caspofungin in a murine model of disseminated caspofungin-resistant candidiasis. Female BALB/c mice were infected with 5 x 10^5^ cells of the echinocandin-resistant *C*. *albicans* clinical isolate (CaLC990) via tail-vein. Caspofungin, DTPA, or the combination was administered intraperitoneally, as indicated, starting 4 hours after infection and then daily for a total of five doses.

Given that DTPA potentiates the activity of caspofungin *in vitro* and also modulates a key virulence trait, we assessed whether there might be therapeutic benefits in a murine model of disseminated candidiasis. Despite the pleiotropic effects of chelators, there is precedent for therapeutic benefits with systemic administration of the broad-spectrum chelator EDTA. EDTA has been shown to enhance the efficacy of the polyene antifungal amphotericin B lipid complex in the treatment of invasive pulmonary aspergillosis in a rat model, with no observed toxicity [73]. Therefore, we tested if a similar dose of DTPA would enhance the therapeutic efficacy of caspofungin in a murine model of systemic infection with a caspofungin-resistant clinical isolate of *C*. *albicans* (Fig 10). Most mice that were infected with 5 x 10^5^ *C*. *albicans* cells succumbed to the infection within 5 days. Treatment with 0.05 mg/kg caspofungin caused a modest improvement in survival (P = 0.1939, Log-Rank Mantel-Cox Test), while treatment with 60 mg/kg DTPA alone did not improve survival. Strikingly, treatment with the combination of caspofungin and DTPA dramatically improved survival compared to treatment with caspofungin alone (P = 0.0296, Log-Rank Mantel-Cox Test). This demonstrates the feasibility of using chelators to enhance echinocandin efficacy *in vivo* in the treatment of echinocandin-resistant infections.

## Discussion

Our results reveal a new mechanism by which metal chelation potentiates echinocandin antifungal activity and modulates a key fungal virulence trait in a leading human fungal pathogen. Based on a screen of 1,280 pharmacologically active compounds, we identified the broad-spectrum chelator DTPA as strongly synergistic with caspofungin against a resistant *C*. *albicans* clinical isolate (Fig 1), and that DTPA enhances echinocandin activity against echinocandin-susceptible and echinocandin-resistant isolates predominantly by chelating magnesium (Fig 2 and S2 Fig). By leveraging a powerful genomic approach to select for mutants resistant to the combination of DTPA and caspofungin coupled with genome sequencing we revealed that mutations in *NIK1* confer resistance to the drug combination (Fig 3). This provides key molecular insight into the mechanism by which DTPA potentiates echinocandin activity, and reveals mutations in a cellular regulator that has not been previously implicated in echinocandin resistance. DTPA activates Hog1, as does caspofungin, and mutations in *NIK1* block Hog1 activation in response to both compounds (Fig 5). Inhibition of (1,3)-β-D-glucan synthesis and activation of Hog1 may provide parallel cell wall insults, such that the combined effect is synergistic. Beyond effects on drug resistance, we discovered that DTPA induces filamentation at 30°C in rich medium in the absence of any inducing cue, and that the effect on morphogenesis is mediated through chelation of zinc (Fig 6). Filamentation induced by DTPA is contingent upon core morphogenetic circuitry including the Ras1-PKA cascade, as well as more specialized morphogenetic regulators, including the transcription factors Rob1 and Brg1 (Figs 7 and 8). This work establishes metal chelation as a powerful strategy to probe circuitry governing cellular responses to drug-induced stress and morphogenesis, and illuminates a new facet to metal homeostasis in modulating host-pathogen interactions.

Metals have complex and crucial roles in all biological systems, where they are incorporated into metalloproteins including enzymes, storage proteins, and transcription factors. They are crucial for survival, but also exert toxicity that is attributable to their capacity to engage in enzyme active sites and potentiate catalytic activity, thus their levels must be tightly controlled [74,75]. As a consequence, mammals exploit strategies to modulate metal availability in order to thwart pathogenic microbes [75]. As both a commensal and opportunistic pathogen, *C*. *albicans* has evolved diverse strategies to ensure metal homeostasis. This is best understood in the context of iron, which is the most abundant transition metal in the human body, and yet its limitation provides a ubiquitous strategy for innate immune defense [75]. In the iron-poor bloodstream, *C*. *albicans* activates iron uptake genes via the transcriptional activator Sef1, which is required for virulence; conversely, in the iron-replete gastrointestinal tract, the transcriptional repressor Sfu1 represses Sef1 at both transcriptional and post-transcriptional levels, thereby dampening expression of iron uptake genes [76,77]. *C*. *albicans* also exploits elaborate systems to sequester iron from the host including the transport of xeno-siderophores via Sit1, and a reductive system to acquire iron from host transferrin proteins or hemoglobin [78,79]. Beyond iron, zinc also has profound impacts on *C*. *albicans* biology and pathogenesis, as well as key roles in host immunity [62,80]. *C*. *albicans* has evolved a zinc acquisition strategy involving secretion of the zinc scavenger Pra1, analogous to siderophore-mediated iron acquisition [62]. Our findings that the broad-spectrum chelator DTPA synergizes with caspofungin and induces filamentation, suggest that bioavailability of metals such as magnesium and zinc in distinct niches within the host may also have a profound impact on fungal morphogenesis and drug resistance.

The pleiotropic effect of metal depletion provides a powerful strategy to probe cellular pathways governing virulence traits and drug resistance. In the context of drug resistance, we illuminate specific circuitry through which DTPA potentiates echinocandin antifungal activity. To date, the principal mechanism of resistance to echinocandins is mutations in the target gene, *FKS1* [14]. We find that either DTPA or magnesium depletion is sufficient to reduce target-based echinocandin resistance. Notably, the concentration of magnesium present in synthetic defined medium is in excess of the concentration of DTPA required to potentiate caspofungin. However, metal cations such as magnesium bind to fungal cell wall components including glucans and mannoproteins [58], limiting the available pool of magnesium that DTPA would need to chelate to potentiate echinocandin activity. Notably, the effects of DTPA on echinocandin activity are blocked by dominant point mutations in the histidine kinase gene *NIK1*. Nik1 likely signals upstream of the Hog1 MAPK cascade, as does the Sln1 histidine kinase in *S*. *cerevisiae* [50,51]. Nik1 is not required for hydrogen peroxide-induced activation of Hog1, nor does deletion of *NIK1* result in constitutive Hog1 activation [81], thus Nik1 is not likely to be the osmosensor or the key histidine kinase responsible for relaying this signal. We establish that mutations in *NIK1* confer increased resistance to caspofungin and DTPA, and impair Hog1 activation in response to these cues (Fig 5). This is consistent with previous findings that deletion of specific *NIK1* HAMP domains impairs Hog1 phosphorylation in response to the fungicide fludioxonil [47]. Nik1 may act as a sensor for specific stresses, such as magnesium depletion. Other histidine kinases, such as the *S*. *cerevisiae* Sln1, have an essential Mg^2+^ ion bound at the active site [82] and the *C*. *albicans* Nik1 may similarly require Mg^2+^. The mutations in *NIK1* may allow for the ion to bind more tightly to the enzyme, or they may induce a conformational change that reduces the necessity of Mg^2+^. Our results support a model in which *NIK1* mutations reduce the dependence of Nik1 on magnesium, thereby blocking the effects of DTPA on echinocandin resistance. Interestingly, deletion of *NIK1* does not confer hypersensitivity to either DTPA or caspofungin (S3 Fig), suggesting that while dominant mutations in *NIK1* are sufficient to confer resistance, DTPA must have additional targets through which it mediates toxicity. This is consistent with previous reports that while Nik1 may have a role in *C*. *albicans* virulence, deletion of *NIK1* does not confer sensitivity to various antifungals [45,83].

**Fig 11:**
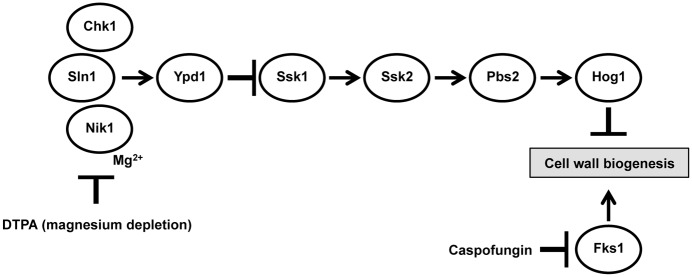
A model depicting the proposed mechanism of action of the combination of DTPA and caspofungin. Treatment with DTPA results in Hog1 activation, in a manner that is blocked by dominant mutations in *NIK1*. Caspofungin impairs cell wall integrity via inhibition of its target, the (1,3)-β-D-glucan synthase Fks1, and also results in Hog1 activation, likely indirectly. Activation of Hog1 by DTPA also impairs cell wall biogenesis; thus, DTPA may act through Nik1 to modulate Hog1 signaling and enhance caspofungin activity.

The resistance phenotypes of the *NIK1* mutants can be explained by the observation that impaired Hog1 signaling leads to resistance to cell wall stressors [53–55], (Fig 4). This is likely due to de-repression of Cek1 kinase activation [54], where Cek1 has roles in cell wall homeostasis [84]. Our findings suggests that DTPA exacerbates the cell wall stress induced by echinocandins by causing further cell wall damage through effects on Hog1 signaling (Fig 11). This finding resonates with previous work illustrating that a natural product that modulates HOG signaling in *A*. *fumigatus* also potentiates caspofungin activity [85]. Together, this reveals new functional relationships governing cell wall stress response that can be targeted to enhance antifungal activity.

Beyond drug resistance, metal chelators also provide a powerful strategy to dissect cellular circuitry governing key virulence traits, such as the transition between yeast and filamentous growth. A striking feature of filamentation induced by DTPA is that it is not contingent upon an increased temperature of 37°C, in contrast to most other cues. The few other cues that are independent of elevated temperature include inhibition of the molecular chaperone Hsp90 [36] and perturbation of cell cycle regulation [86–88]; filamentation induced by these cues is independent of the key transcriptional regulator of morphogenesis, Efg1 [36,86,87]. In contrast, filamentation induced by DTPA is contingent upon Efg1 (Fig 7A), suggesting that metal chelation does not induce morphogenesis by perturbing Hsp90 function or cell cycle progression. Filamentation induced by DTPA is dependent not only on Efg1, but also on upstream PKA signaling components including Ras1, Cyr1, and the PKA catalytic subunits (Fig 8B). Ras1-PKA signaling is also required to down regulate expression of the transcriptional repressor gene *NRG1* upon initiation of filamentous growth [70,72], and we observe a pronounced reduction of Nrg1 levels in filaments induced by diverse cues including DTPA (Fig 8A). Depletion of Nrg1 may also be due to post-translation control, as the E3 ubiquitin ligase Ubr1 regulates degradation of the transcriptional repressor Cup9, thereby enabling transcriptional activation of the kinase gene *SOK1* and Nrg1 degradation upon release from the quorum sensing molecule farnesol at 37°C [72]. We find that Sok1 is required for filamentation induced by DTPA but Ubr1 is dispensable (Fig 8C), as Nrg1 protein levels are reduced in DTPA-induced filaments even in the absence of Ubr1 (Fig 9). This suggests that DTPA may impair Cup9 function, thereby inducing Nrg1 degradation and bypassing the requirement for Ubr1. Thus, our results highlight functional divergence in the circuitry governing post-translational regulation of Nrg1 in response to different filament-inducing cues.

There is additional regulatory complexity in the circuitry through which metal chelation induces morphogenesis. We established that DTPA induces filamentation via depletion of zinc, and that this requires not only core morphogenetic signaling through the core morphogenetic Ras1-PKA cascade and through Nrg1, but also requires the biofilm transcriptional regulators, Brg1 and Rob1 (Fig 7). This functional relationship resonates with additional connections between zinc homeostasis and biofilm maturation. For example, Zap1 is a zinc-responsive transcription factor that regulates expression of several genes involved in biofilm matrix production [89]. Deletion of *ZAP1* causes filamentation defects [61] and an increase in the proportion of yeast cells in biofilms [89,90]. Further, overexpression of the zinc transporter *ZRT2* in strains lacking Zap1 restores expression of a yeast-specific reporter to wild-type levels [90]. Our finding that DTPA induces filamentation via depletion of zinc in a manner that depends upon Rob1, Brg1, Ras1-PKA signaling, and Nrg1 degradation expands our appreciation of the mechanisms through which metals modulate morphogenesis (Figs 7–9). Consistent with our results, exogenous zinc has been observed to suppress *C*. *albicans* filamentation [91,92], though the mechanisms involved have remained enigmatic. Our results suggest that zinc modulates complex cellular circuitry governing a key fungal virulence trait.

Perturbing metal homeostasis has broad therapeutic potential for the treatment of infectious disease. Iron chelators such as ciclopirox olamine provide effective therapy for superficial mycoses [40,41], and have evaded resistance despite decades of clinical use [41]. Broad spectrum chelators have also shown promise as therapeutic agents in animal models. EDTA is structurally related to DTPA, and has therapeutic benefit in a mouse model of *Pseudomonas aeruginosa* induced pneumonia [93]. EDTA also enhances the efficacy of polyenes in a rat model of invasive pulmonary aspergillosis, with no observed toxicity [73]. Similarly, we observe a drastic increase in the efficacy of caspofungin in combination with DTPA in a murine model of caspofungin-resistant disseminated candidiasis, demonstrating the potential utility of metal chelation as a therapeutic strategy. However, exploiting the therapeutic potential of metal limitation will likely require strategies to enhance selectivity for the pathogen and thereby minimize deleterious effects on the host. This could be achieved by targeting the pathogen’s metal import or homeostasis systems. With strategically designed high-throughput screens for molecules that target metal homeostasis [94], there is considerable promise for discovering novel classes of antimicrobial agents. It is exquisitely clear that metal homeostasis has profound impacts on susceptibility to infectious disease, as patients with iron overload conditions due to frequent transfusions are vulnerable to infection, as are patients with inherited or acquired iron storage disorders such as those with haemochromatosis [75]. Although haemochromatosis is associated with increased susceptibility to infection with enteric Gram-negative pathogens including *Vibrio vulnificus* and *Yersinia enterocolitica*, they also have iron-deficient macrophages and consequently are more resistant to intracellular pathogens such as *Salmonella enterica* subsp. *enterica* serovar Typhi and *Mycobacterium tuberculosis* [95–98]. There is a growing appreciation that other metals also have a tremendous impact on microbial virulence and host immunity. Defining the mechanisms that govern metal homeostasis in hosts and pathogens, and the circuitry through which metals control key virulence traits holds great promise for revealing new therapeutic strategies for life-threatening infectious disease.

## Materials and Methods

### Ethics statement

All procedures for animal research were approved by the Institutional Animal Care and Use Committee (IACUC protocol A114-14-05) at Duke University according to the guidelines of the Animal Welfare Act, The Institute of Laboratory Animal Resources Guide for the Care and Use of Laboratory Animals, and Public Health Service Policy.

### Culture conditions

Archives of *C*. *albicans* strains were maintained at −80°C in rich medium (YPD: 1% yeast extract, 2% bactopeptone, 2% glucose, with 2% agar for solid medium) with 25% glycerol. Strains were grown in YPD, synthetic defined (SD) medium or RPMI medium. SD was prepared as follows: 0.17% yeast nitrogen base without ammonium sulfate, 0.1% glutamic acid, 2% glucose, supplemented with arginine HCl (50 mg/L) and histidine HCl (20 mg/L) as required, pH 6. RPMI was prepared as follows: 10.4 g/L RPMI-1640, 3.5% MOPS, 2% glucose, supplemented with an additional 5 mg/mL histidine as required, pH 7. Spider Medium and Lee’s Medium were prepared as previously described [99,100].

Chelex 100 resin (BioRad) was used to deplete SD medium of its metal components. For the growth assay, 2.5 g of resin was added per 100 mL of medium (except for Fig 3C, where 2.0 g was used), and for filamentation assays, 2.0 g of resin per 100 mL. The medium with the resin stirred for 1.5 hours at room temperature and was filtered sterilized. Metals were added back to the following concentrations, as indicated: CuSO_4_ 80 μg/L; FeCl_3_ 400 μg/L; MnSO_4_ 800 μg/L; ZnSO_4_ 800 μg/L; MgSO_4_ 1 g/L; CaCl_2_ 0.2 g/L.

### Strains

All strains used in this study are listed in S1 Table. All oligonucleotide sequences used in this study are included in S2 Table. To select for nourseothricin (NAT)-resistant mutants, NAT (Jena Bioscience) stock solution was prepared in water at a concentration of 250 mg/mL and YPD plates were supplemented with 150 μg/mL NAT.

### CaLC3841

To introduce the *NIK1*^G2048T^ mutation into a wild-type strain, the plasmid pLC889 was digested with BssHII and transformed into CaLC239. For NAT-resistant transformants, proper integration was tested by PCR with primers oLC3508/oLC275 and oLC3515/oLC274. The *SAP2* promoter was induced to drive expression of FLP recombinase to excise the NAT marker cassette [101,102]. Presence of the *NIK1*^G2048T^ allele and absence of spurious mutations was verified by amplifying the region with the mutation (oLC3508/oLC275) and sequencing with oLC3511.

### CaLC3872

To introduce the *NIK1*^*G1319C*^ allele into the caspofungin-resistant clinical isolate (DPL15), the plasmid pLC891 was digested with BssHII and transformed into CaLC990. For NAT-resistant transformants, proper integration was tested by PCR with primer pairs oLC3508/oLC275 and oLC3515/oLC274. The *SAP2* promoter was induced to drive expression of FLP recombinase to excise the NAT marker cassette. The presence of the *NIK1*^*G1319C*^ allele was verified by amplifying the region with the mutation (oLC3508/oLC275) and sequencing with oLC3703.

### CaLC3886

To introduce the *YKE2*^*T95A*^ mutation into a wild-type strain, the plasmid pLC907 was digested with BssHII and transformed into CaLC239 using standard protocols. For NAT-resistant transformants, proper integration was tested by PCR with primers oLC3502/oLC275. The *SAP2* promoter was induced to drive expression of FLP recombinase to excise the NAT marker cassette. Presence of the *YKE2*^*T95A*^ allele and absence of spurious mutations was verified by amplifying the region with the mutation (oLC3502/oLC275) and sequencing with oLC3503.

### CaLC4199

To introduce the *NIK1*^G2048T^ mutation into the *hog1Δ/hog1Δ* mutant, the plasmid pLC889 was digested with BssHII and transformed into CaLC3943. For NAT-resistant transformants, proper integration was tested by PCR with primers oLC3508/oLC275 and oLC3515/oLC274. The *SAP2* promoter was induced to drive expression of FLP recombinase to excise the NAT marker cassette. Presence of the *NIK1*^G2048T^ allele and absence of spurious mutations was verified by amplifying the region with the mutation (oLC3508/oLC3512) and sequencing with oLC3511. Absence of a wild-type allele of *HOG1* was verified using oLC4151/oLC4152.

### CaLC4530

The *SSK1* knockout construct was PCR-amplified from pLC49 using primer pair oLC4427/oLC4428, containing sequence homologous to upstream and downstream regions of *SSK1*, and transformed into the wild type CaLC2302. NAT-resistant transformants were PCR tested for proper integration of the construct using primer pairs oLC275/oLC4429 and oLC274/oLC4431. The *SAP2* promoter was induced to drive expression of FLP recombinase [101,102] to excise the NAT marker cassette. The second *SSK1* allele was deleted in the same manner. Absence of a wild-type allele of *SSK1* was verified using oLC4429 and oLC4430.

### CaLC2367

To C-terminally 3xHA tag Nrg1, the construct was amplified from pFA-*ARG4* in which *NRG1* and HA were cloned at the BamHI and SalI sites, with primers oLC4581 and oLC4582. The construct was transformed into CaLC192 and arginine protrophs were tested for proper integration by PCR with primer pairs oLC4583/oLC4584.

### CaLC4453

To C-terminally HA tag Nrg1 in a wild type, the construct was amplified from pLC576 [103] with primers oLC4370 and oLC4371 and transformed into CaLC2302. Arginine protrophs were tested for proper integration by PCR with primer pairs oLC4372/oLC4374 and oLC4375/oLC4373.

### CaLC4454

To C-terminally HA tag Nrg1 in a *ubr1*Δ*/ubr1*Δ mutant, the construct was amplified from pLC576 with primers oLC4370 and oLC4371 and transformed into CaLC4455. Arginine protrophs were tested for proper integration by PCR with primer pairs oLC4372/oLC4374 and oLC4375/oLC4373. Absence of a wild-type *UBR1* allele was verified with primers oLC4240 and oLC4241.

### Plasmid construction

All oligonucleotide sequences used in this study are included in S2 Table.

### pLC889

Downstream of *NIK1* was amplified by PCR from CaLC239 genomic DNA using primers oLC3513/oLC3514. This PCR product and pLC49 [25] were digested with SacI and SacII and ligated. Integration was verified by sequencing with oLC274. *NIK1*^G2048T^ was amplified by PCR from CaLC3350 genomic DNA using primers oLC3509/oLC3512. This PCR product and the plasmid pLC49+CaLC239 oLC3513/oLC3514 (above) were digested with KpnI and ApaI and ligated. Presence of the *NIK1*^G2048T^ allele and lack of spurious mutations was verified by sequencing with primers oLC243, oLC3703, oLC3704, oLC3511, oLC3785, oLC3786, oLC3787 and oLC3788.

### pLC891

Downstream of *NIK1* was amplified by PCR from CaLC239 genomic DNA using primers oLC3513/oLC3514. This PCR product and pLC49 were digested with SacI and SacII and ligated. Integration was verified by sequencing with oLC274. The *NIK1*^*G1319C*^ allele was amplified by PCR from CaLC3161 genomic DNA using primers oLC3509/oLC3512. This PCR product and the plasmid pLC49+CaLC239 oLC3513/oLC3514 (above) were digested with KpnI and ApaI and ligated. Presence of the *NIK1*^*G1319C*^ allele and lack of spurious mutations was verified by sequencing with primers oLC243, oLC3703, oLC3704, oLC3511, oLC3785, oLC3786, oLC3787 and oLC3788.

### pLC907

Downstream of *YKE2* was amplified by PCR from CaLC239 genomic DNA using primers oLC3505/oLC3506. This PCR product and pLC49 were digested with SacI and SacII and ligated. Integration was verified by sequencing with oLC274. *YKE2*^*T95A*^ was amplified by PCR from CaLC3211 genomic DNA using primers oLC3503/oLC3504. This PCR product and the plasmid pLC49+CaLC239 oLC3505/oLC3506 (above) were digested with KpnI and ApaI and ligated. Presence of the *YKE2*^*T95A*^ allele and lack of spurious mutations was verified by sequencing with primers oLC243 and oLC275.

### Minimum inhibitory concentration and checkerboard assays

Resistance to single antifungal drugs or drug combinations was assayed in 96-well microtiter plates (Sarstedt) as previously described [20,21,104]. Assays were performed in a total volume of 0.2 mL/well with 2-fold dilutions of each drug in the indicated medium. Plates were incubated in the dark at 30°C before OD_600_ were determined using a spectrophotometer (Molecular Devices), at the indicated time point. Data was displayed as heat maps using Java TreeView 1.1.6.

DTPA (Sigma) was dissolved in NaOH and ddH_2_O and the pH was adjusted to 7. DPI (Sigma) and Ciclopirox ethanolamine (ShelleckChem) were prepared in DMSO. MRS2159 (Sigma) was prepared in ddH_2_O. NPA (Sigma) was prepared in methanol. Caspofungin was a generous gift from Merck and was dissolved in ddH_2_O.

### Whole genome sequencing

Cell pellets for whole genome sequencing were prepared by centrifuging 50 mL of overnight culture at 3,000 rpm for 10 minutes and washing with 40 mL of ddH_2_O. Sequencing libraries were prepared and alignment performed as previously described [105]. The sequence data is publicly available on the NCBI Sequence Read Archive with accession number SRX1799649.

### Quantitative reverse transcription-PCR (qRT-PCR)

To monitor expression of *BRG1* and *ROB1*, the strains were grown overnight in YPD at 30°C, diluted to an OD_600_ of 0.1 in the presence or absence of 100 μM DTPA, and grown for 24 hours at 30°C. Subsequently, untreated cells were diluted to an OD_600_ of 0.1 in YPD and DTPA-treated cells were diluted into YPD containing 100 μM DTPA and grown for 4 hours. Cultures were pelleted and frozen at -80°C. RNA extraction, complementary DNA synthesis and PCR were performed as previously described [106]. Reactions were performed in triplicate, for two biological replicates using the primers, oLC2635/oLC2636 (*BRG1*), oLC2637/oLC2638 (*ROB1*), oLC2285/oLC2286 (*ACT1*) and oLC752/oLC753 (*GPD1*). All data were normalized to *ACT1* and *GPD1*. Data were analyzed using the BioRad CFX Manager 3.1. To measure expression of *HWP1*, *ZRT1* and *ZRT2*, the strains were grown overnight in YPD at 30°C, diluted to an OD_600_ of 0.1 in the presence or absence of 10 μM DTPA, and the presence or absence of 20 μg/mL doxycycline and grown for 24 hours at 30°C. Subsequently, cells were diluted to an OD_600_ of 0.05 in YPD with or without 20 μg/mL doxycycline and with or without 50 μM DTPA. Cells were pelleted for RNA extraction after 4.5 hours of growth at 30°C. Analysis was performed as above, using the primers oLC752/oLC753 (*GPD1*), oLC3796/oLC751 (*HWP1*), oLC5109/oLC5115 (*ZRT1*) and oLC5111/oLC5112 (*ZRT2*). Data was normalized to *GPD1*. Oligonucleotide sequences are included in S2 Table.

### Microscopy

Imaging from liquid media was performed using differential interference contrast microscopy using a Zeiss Axio Imager.MI (Carl Zeiss). TPEN (N,N,N,N-Tetrakis(2-pyridylmethyl)ethylenediamine) (Sigma) was prepared in ethanol. Geldanamycin (LC laboratories, G-4500) was dissolved in DMSO.

### Cell staining

A caspofungin-resistant isolate (CaLC990) was grown for 16 hours in RPMI in the presence or absence of 0.32 μg/mL caspofungin or 50 μM DTPA. Cells were washed with PBS and stained with Aniline Blue (0.05%), Calcofluor White (25 μg/mL) or Concanavalin A (20 μg/mL). Images for a single stain were taken at the same exposure.

### Protein extraction and western blot analysis

To test for Mkc1 activation via phosphorylation, *C*. *albicans* was initially grown overnight at 30°C in YPD while shaking at 200 rpm. Subsequently, the stationary phase culture was split and adjusted to an OD_600_ of 0.1 in 2 mL YPD with or without 100 μM DTPA and grown as above for 24 hours. These stationary subcultures were each split into two and adjusted to an OD_600_ of 0.1 in 50 mL YPD. These were grown for 2.5 hours at 30°C, at which point each condition was treated with 125 ng/mL caspofungin for 1 hour. Cells were harvested by centrifugation and washed once with ice-cold 1×PBS. Pellets were resuspended in 200 μl lysis buffer (50 mM HEPES pH 7.5, 150 mM NaCl, 5 mM EDTA, 1% Triton X100, protease inhibitor cocktail (Roche Diagnostics), 50 mM NaF, 10 mM Na_3_VO_4_, 1 mM PMSF). Proteins were extracted and analyzed as previously described [21]. Proteins were separated by 8% SDS-PAGE and blocked with 5% bovine serum albumin in tris-buffered saline with 0.1% tween. Blots were hybridized with antibodies against α-phospho-p44/42 MAPK (Thr202/Tyr204) (7:10,000, Cell Signaling) and α-PSTAIRE (1:5,000; Sigma).

To test for Hog1 activation via phosphorylation, cells were initially grown as above. Stationary phase cultures were split and adjusted to an OD_600_ of 0.1 in 50 mL RPMI and grown for 5 hours 50 minutes at 30°C. For DTPA-treated cultures, DTPA was added at time 0. For caspofungin-treated cultures, cells were grown for 4 hours 20 minutes, treated with caspofungin, and were left to grow for an additional 1 hour 30 minutes. For H_2_O_2_-treated cultures, 10 mM H_2_O_2_ was added for the last 10 minutes of growth. Cells were harvested and proteins were analyzed as above. Blots were hybridized with antibodies against α-phospho-p38 MAPK (T180/Y182) (1:2,500, Cell Signaling), α-Hog1 (1:1,2500, AbDseroTec), and α-tubulin (1,1000, Santa Cruz Biotechnology). To test for Hog1 activation in response to magnesium depletion, wild-type cells (CaLC239), were grown in 10 mL of SD medium either in the presence of 20 μM DTPA, or in Chelex-treated SD with all metals restored except for magnesium. Cells were grown for 5 hours. Proteins were extracted and analyzed as above.

To monitor total levels of Nrg1, strains were cultured as described above and diluted to an OD_600_ of 0.1 in 10 mL of the appropriate media (Fig 8) or 25 mL YPD (Fig 9) to grow for 4.5 hours. Protein was extracted as previously described [22], and analyzed as above but blocked with 5% skim milk in phosphate-buffered saline. Blots were hybridized with antibodies against α-HA (1:5000, Roche Diagnostics).

### Murine model of systemic infection

Female BALB/c mice were inoculated with 5x10^5^ cells of *C*. *albicans* strain CaLC990, resuspended in 150 μl of sterile PBS, by tail vein injection. There were four treatment groups consisting of ten mice each, which were ordered from Charles River. Test groups were treated with vehicle (water), DTPA (Sigma-Aldrich) alone at 60 mg/kg/dose, caspofungin (Merck) alone at 0.05 mg/kg/dose, and a combination of DTPA and caspofungin at these doses. Drug doses were administered by intraperitoneal injection starting four hours post-infection, then every 24 hours for a total of five doses. Mice were monitored daily for weight loss and overall health condition, and were euthanized upon reaching humane endpoints as defined in Duke IACUC protocol A114-14-05. The survival curve was calculated using Prism software version 4.

## Supporting Information

S1 Fig Validation of compounds identified as hits in the initial screen. All compounds were tested in a secondary dose response assay by testing their efficacy in combination with caspofungin, except for rac-2-Ethoxy-3-hexadecanamido-1-propylphosphocholine, rac-2-Ethoxy-3-octadecanamido-1-propylphosphocholine, Tamoxifen citrate salt (all of which inhibit protein kinase C), and farnesyl thiosalicylic acid (due to lack of compound). Assays were performed in RPMI medium in the presence or absence of a fixed concentration of caspofungin (8 μg/mL), as indicated. Data was analyzed after 96 hours at 30°C, and normalized as in Fig 1.
(TIFF)

S2 Fig DTPA enhances caspofungin activity against a wild-type strain via depletion of magnesium. Chelex 100 resin was used to deplete synthetic defined medium of its metal components, as in Fig 2B. Addition of magnesium to metal-depleted medium best restores growth of a wild-type strain (SN95) in caspofungin (P<0.001, two-way ANOVA, Bonferroni correction). Data are means ± SD for triplicate samples.
(TIFF)

S3 Fig Point mutations in *YKE2* or *NIK1* confer resistance to the combination of DTPA and caspofungin in a wild-type laboratory strain. **A)** Introduction of the *YKE2*^T95A^ allele or the *NIK1*^G2048T^ allele into a wild-type strain (SN95) confers resistance to DTPA and caspofungin. Checkerboard analysis was performed as in Fig 3. **B)** Deletion of *NIK1* does not alter susceptibility to either DTPA or caspofungin. Assays were performed in RPMI and data was analyzed after 72 hours at 30°C, and normalized as in Fig 1.
(TIFF)

S4 Fig DTPA does not activate the cell wall integrity pathway nor does it alter cell wall architecture. **A)** Wild-type cells (SN95) were initially grown in the presence or absence of 100 μM DTPA in YPD at 30°C for 24 hours. Cells were subcultured into YPD with or without 100 μM DTPA and grown for 2.5 hours at 30°C, at which point they were either left untreated or treated with 125 ng/mL caspofungin for 1 hour. Total protein was resolved by SDS-PAGE and blots were hybridized with α-phospho p44/42 MAPK to monitor phosphorylated Mkc1 and α-PSTAIRE (Cdc28) as a loading control. **B)** Treatment with DTPA does not result in significant changes to the cell wall architecture, as does caspofungin. A caspofungin-resistant clinical isolate (CaLC990) was grown in RPMI and treated with 50 μM DTPA or 0.32 μg/mL caspofungin for 16 hours. Cells were stained with Aniline Blue to measure glucans, Calcofluor White to measure chitin or Concanavalin A to measure mannans. Scale bar is 20 μM.
(TIFF)

S5 Fig Metal-depletion, zinc-depletion, or treatment with DTPA all induce filamentation. Metal-depletion, zinc-depletion or treatment with DTPA significantly increase filamentation relative to the complete synthetic medium (one-way ANOVA, Bonferroni correction). Restoring all metals, or zinc alone, or addition of excess zinc to DTPA-treated cells all significantly block filamentation (one-way ANOVA, Bonferroni correction). (*** P≤0.0001, ** P≤0.0004, * P≤0.0015). Filamentation was quantified by counting the percentage of filamentous cells in three fields of view (at least 180 cells).
(TIFF)

S6 Fig Depletion of zinc best induces filamentous growth of *C*. *albicans*. **A)** Wild-type cells (SN95) were grown in untreated synthetic defined medium, or that treated with Chelex 100 resin. All metals were added back except for individual metals, as indicated. Cells were grown for 24 hours at 30°C. Scale bar is 50 μM. **B)** Filamentation was quantified by counting the percentage of filamentous cells in four fields of view (at least 250 cells). Only depletion of all metals or of zinc alone induced filamentation significantly higher than complete medium (*** P<0.0001, one-way ANOVA, Dunnett’s test).
(TIFF)

S7 Fig The *brg1*Δ*/brg1*Δ and *rob1*Δ*/rob1*Δ mutants are able to filament in response to multiple inducing cues. Untreated and geldanamycin (GdA)-treated cells were grown in YPD at 30°C for 24 hours. Cells were grown in the other conditions, as indicated, for 24 hours. Scale bar is 20 μM.
(TIFF)

S8 Fig Cells treated with DTPA to monitor Nrg1 levels are filamentous. The cells grown for protein extraction and Western blot analysis (Fig 9) were imaged at 4.5 hours. Scale bar is 20 μM.
(TIFF)

S1 Table Strains used in this study. (DOCX)

S2 Table Oligonucleotides used in this study. (DOCX)
