# Supplementary figures and images for: Metal Chelation as a Powerful Strategy to Probe Cellular Circuitry Governing Fungal Drug Resistance and Morphogenesis
Source: PLoS Genet. 2016 Oct 3;12(10):e1006350. doi: 10.1371/journal.pgen.1006350 (PMC5047589; doi:10.1371/journal.pgen.1006350)

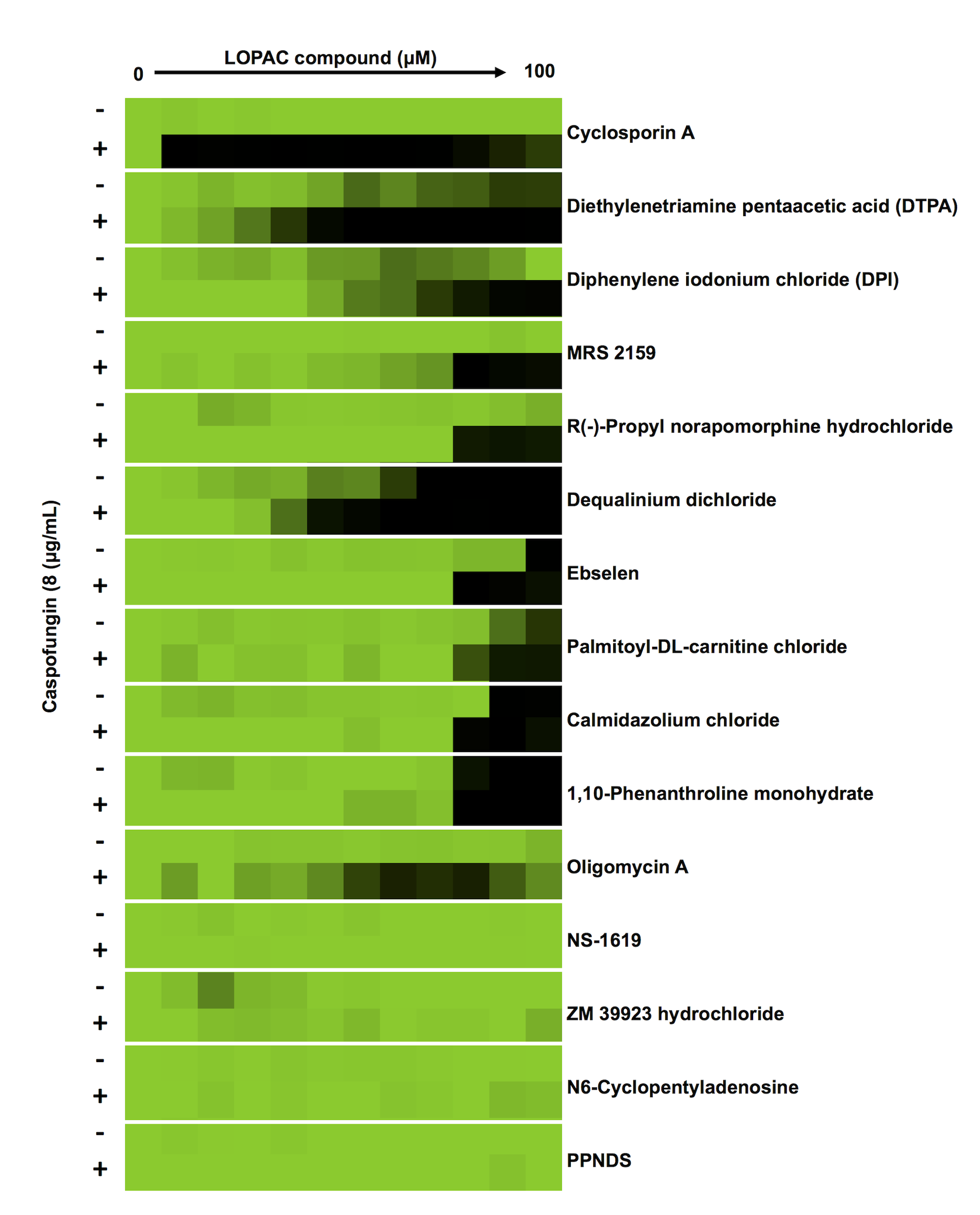

Supplement: S1 Fig — All compounds were tested in a secondary dose response assay by testing their efficacy in combination with caspofungin, except for rac-2-Ethoxy-3-hexadecanamido-1-propylphosphocholine, rac-2-Ethoxy-3-octadecanamido-1-propylphosphocholine, Tamoxifen citrate salt (all of which inhibit protein kinase C), and farnesyl thiosalicylic acid (due to lack of compound). Assays were performed in RPMI medium in the presence or absence of a fixed concentration of caspofungin (8 μg/mL), as indicated. Data was analyzed after 96 hours at 30°C, and normalized as in Fig 1. (TIFF) [file pgen.1006350.s001.tiff]

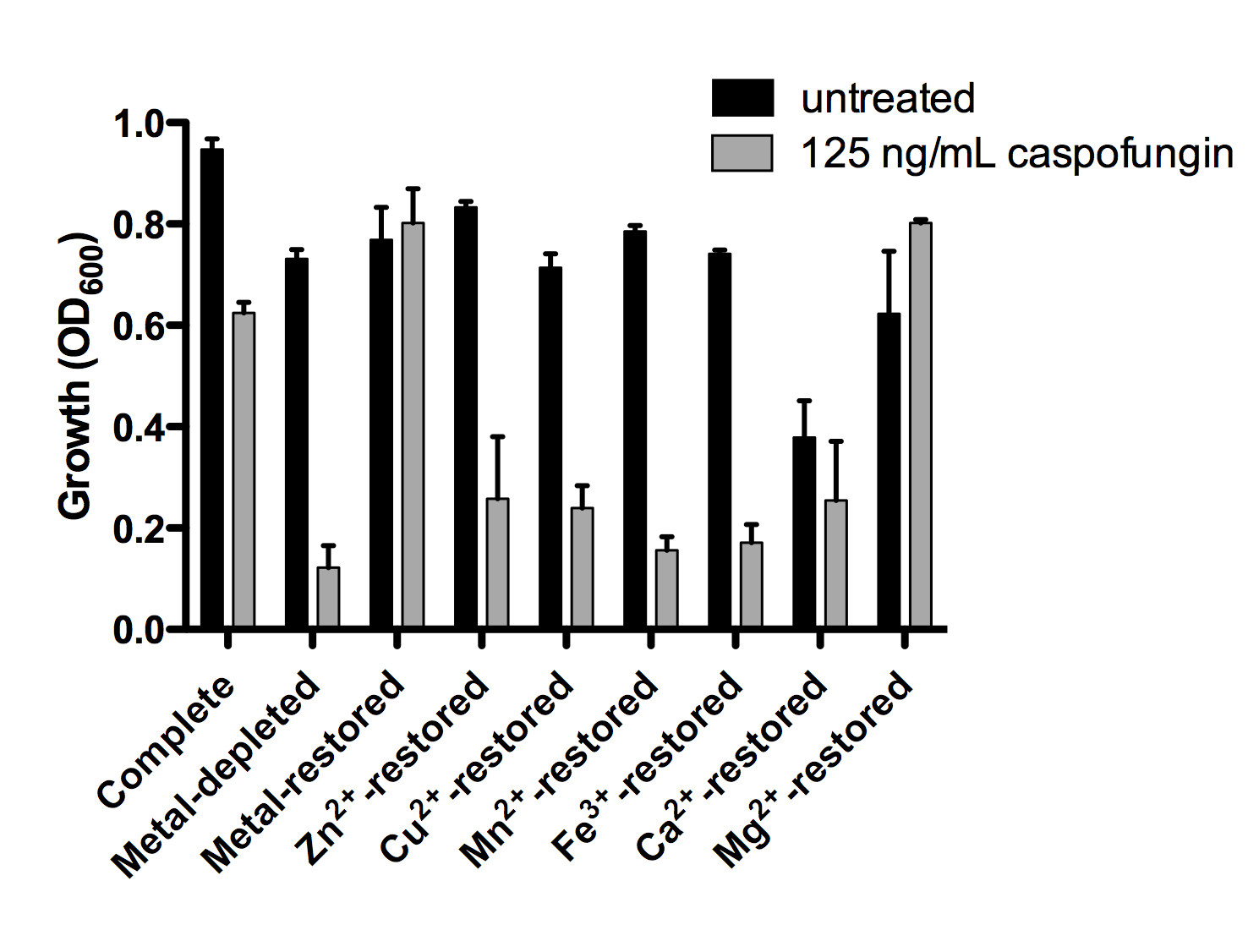

Supplement: S2 Fig — Chelex 100 resin was used to deplete synthetic defined medium of its metal components, as in Fig 2B. Addition of magnesium to metal-depleted medium best restores growth of a wild-type strain (SN95) in caspofungin (P<0.001, two-way ANOVA, Bonferroni correction). Data are means ± SD for triplicate samples. (TIFF) [file pgen.1006350.s002.tiff]

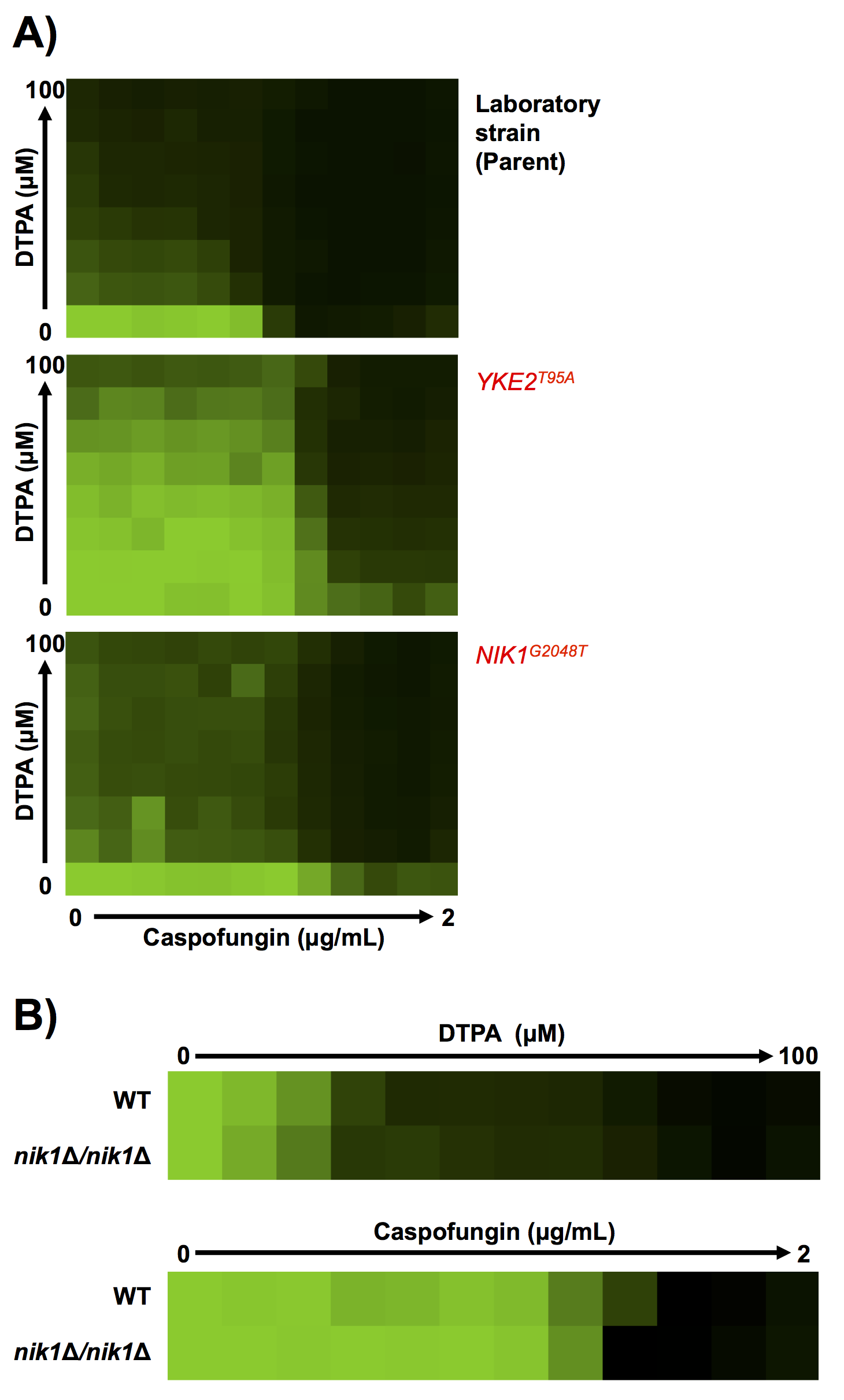

Supplement: S3 Fig — A) Introduction of the YKE2T95A allele or the NIK1G2048T allele into a wild-type strain (SN95) confers resistance to DTPA and caspofungin. Checkerboard analysis was performed as in Fig 3. B) Deletion of NIK1 does not alter susceptibility to either DTPA or caspofungin. Assays were performed in RPMI and data was analyzed after 72 hours at 30°C, and normalized as in Fig 1. (TIFF) [file pgen.1006350.s003.tiff]

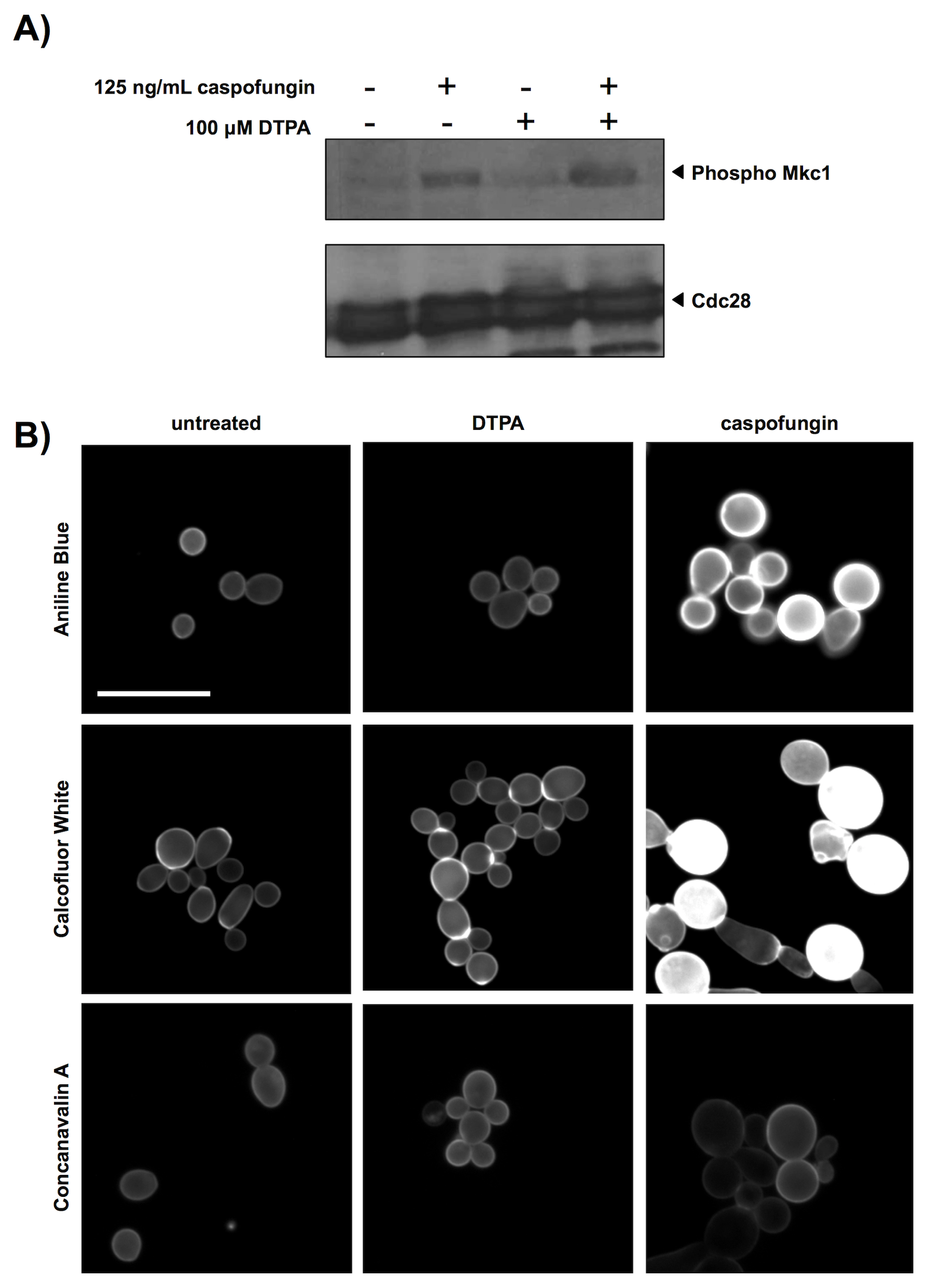

Supplement: S4 Fig — A) Wild-type cells (SN95) were initially grown in the presence or absence of 100 μM DTPA in YPD at 30°C for 24 hours. Cells were subcultured into YPD with or without 100 μM DTPA and grown for 2.5 hours at 30°C, at which point they were either left untreated or treated with 125 ng/mL caspofungin for 1 hour. Total protein was resolved by SDS-PAGE and blots were hybridized with α-phospho p44/42 MAPK to monitor phosphorylated Mkc1 and α-PSTAIRE (Cdc28) as a loading control. B) Treatment with DTPA does not result in significant changes to the cell wall architecture, as does caspofungin. A caspofungin-resistant clinical isolate (CaLC990) was grown in RPMI and treated with 50 μM DTPA or 0.32 μg/mL caspofungin for 16 hours. Cells were stained with Aniline Blue to measure glucans, Calcofluor White to measure chitin or Concanavalin A to measure mannans. Scale bar is 20 μM. (TIFF) [file pgen.1006350.s004.tiff]

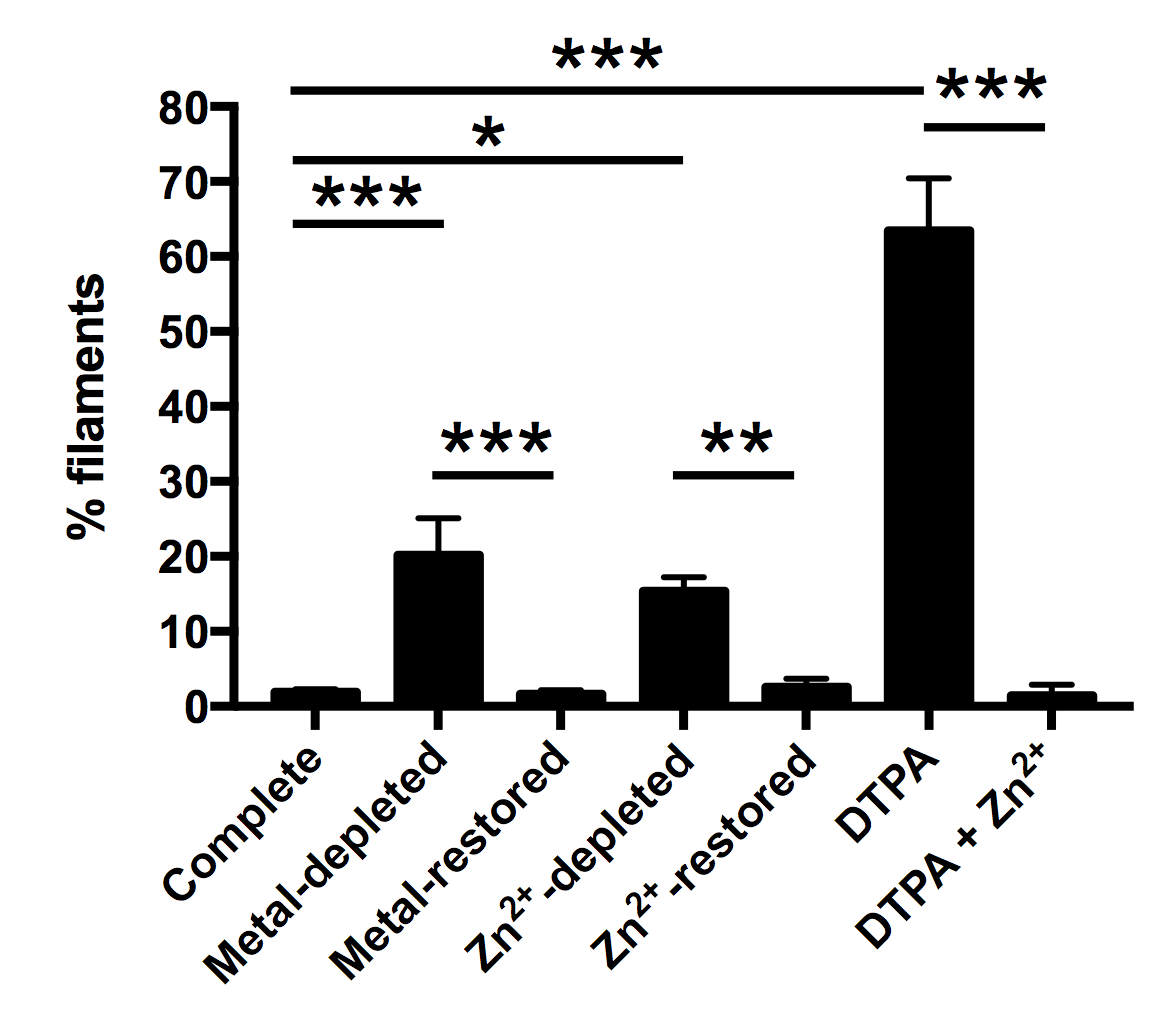

Supplement: S5 Fig — Metal-depletion, zinc-depletion or treatment with DTPA significantly increase filamentation relative to the complete synthetic medium (one-way ANOVA, Bonferroni correction). Restoring all metals, or zinc alone, or addition of excess zinc to DTPA-treated cells all significantly block filamentation (one-way ANOVA, Bonferroni correction). (*** P≤0.0001, ** P≤0.0004, * P≤0.0015). Filamentation was quantified by counting the percentage of filamentous cells in three fields of view (at least 180 cells). (TIFF) [file pgen.1006350.s005.tiff]

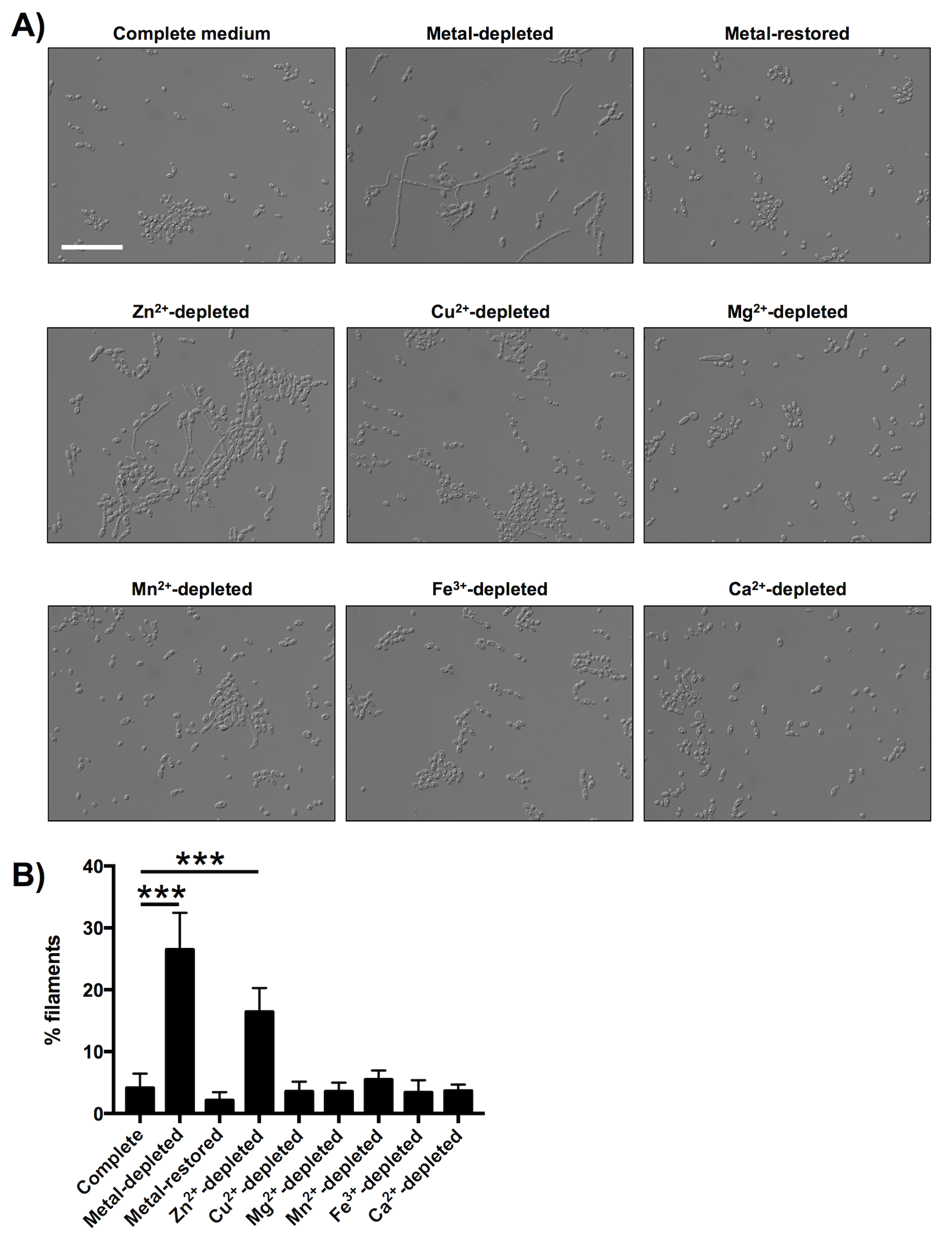

Supplement: S6 Fig — A) Wild-type cells (SN95) were grown in untreated synthetic defined medium, or that treated with Chelex 100 resin. All metals were added back except for individual metals, as indicated. Cells were grown for 24 hours at 30°C. Scale bar is 50 μM. B) Filamentation was quantified by counting the percentage of filamentous cells in four fields of view (at least 250 cells). Only depletion of all metals or of zinc alone induced filamentation significantly higher than complete medium (*** P<0.0001, one-way ANOVA, Dunnett’s test). (TIFF) [file pgen.1006350.s006.tiff]

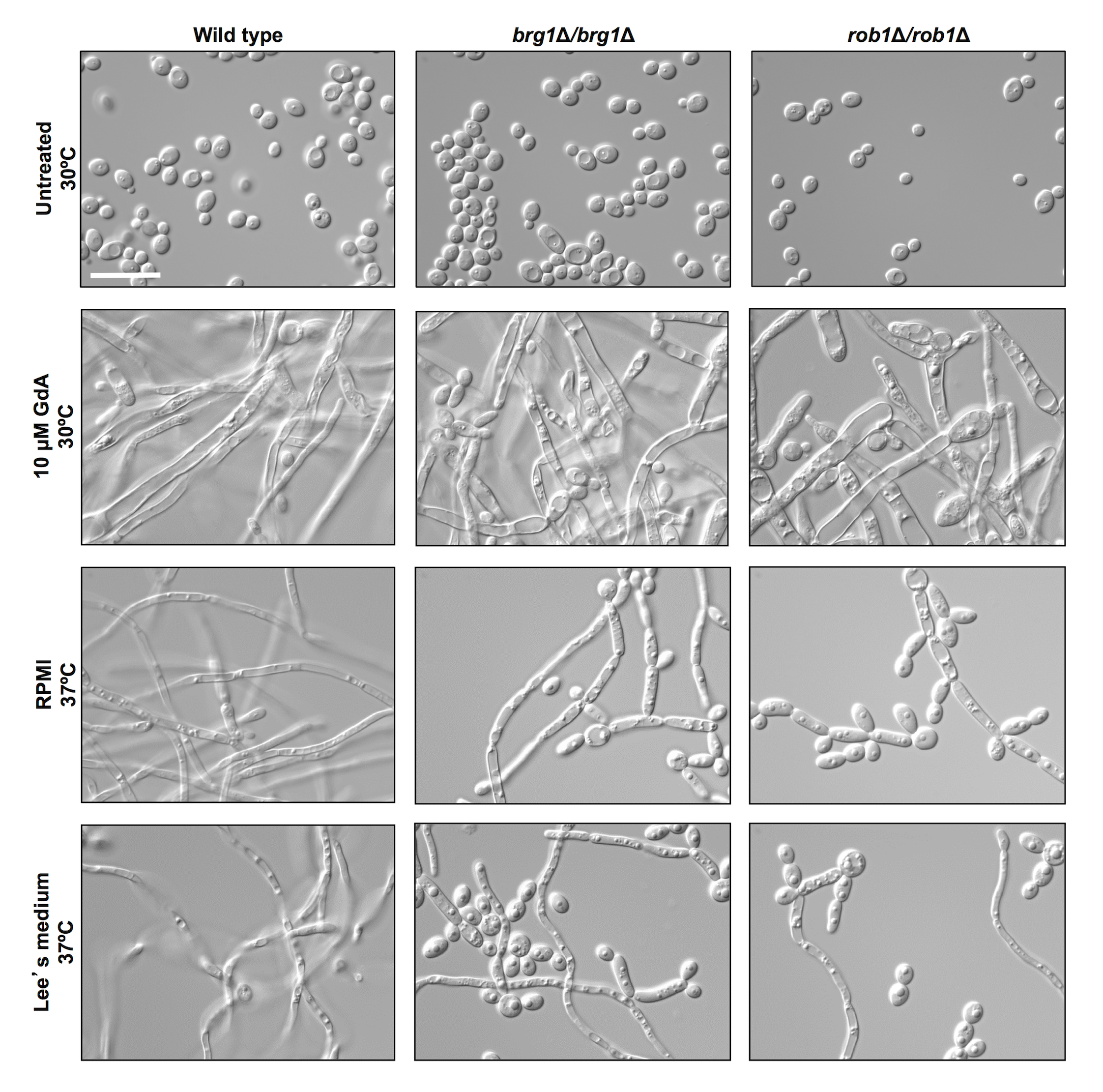

Supplement: S7 Fig — Untreated and geldanamycin (GdA)-treated cells were grown in YPD at 30°C for 24 hours. Cells were grown in the other conditions, as indicated, for 24 hours. Scale bar is 20 μM. (TIFF) [file pgen.1006350.s007.tiff]

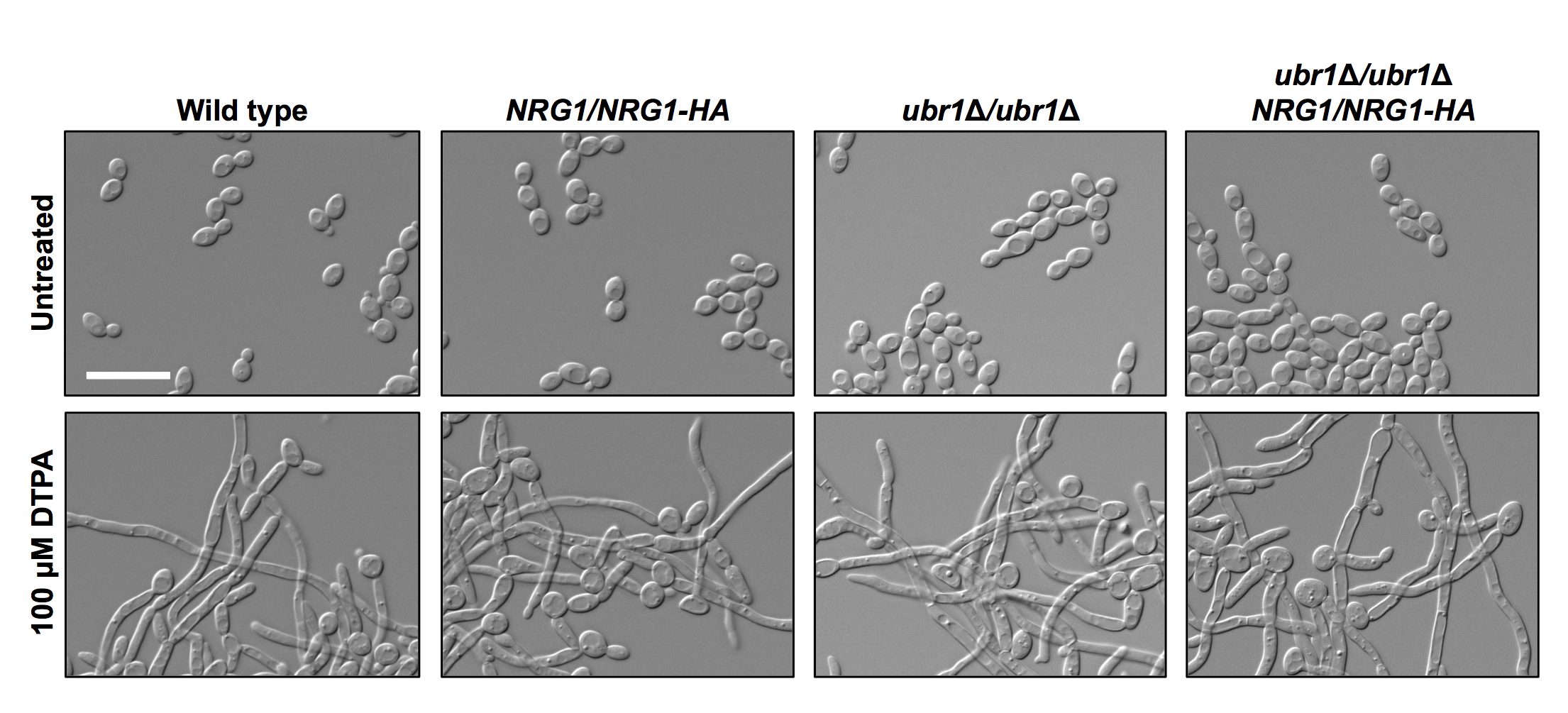

Supplement: S8 Fig — The cells grown for protein extraction and Western blot analysis (Fig 9) were imaged at 4.5 hours. Scale bar is 20 μM. (TIFF) [file pgen.1006350.s008.tiff]
