# Supplementary material for: Metal Chelation as a Powerful Strategy to Probe Cellular Circuitry Governing Fungal Drug Resistance and Morphogenesis
Source: PLoS Genet. 2016 Oct 3;12(10):e1006350. doi: 10.1371/journal.pgen.1006350 (PMC5047589; doi:10.1371/journal.pgen.1006350)
Supplement: S1 Table — (DOCX) [file pgen.1006350.s009.docx]

**S1 Table.** Strains used in this study.

| **Name** | **Genotype** | **Source** |
| --- | --- | --- |
| CaLC990 (DPL15) | Clinical isolate,  *FKS1^T1922C^*/*FKS1^T1922C^* | [1] |
| CaLC239 (SN95) | *arg4*∆*/arg4*∆ *his1*∆*/his1*∆ *URA3/ura3*∆*::imm^434^ IRO1/iro1*∆*::imm434* | [2] |
| CaLC3211  (resistant mutant #1) | As SN95, *YKE2/YKE2^T95A^* | This study |
| CaLC3350  (resistant mutant #2) | As SN95*, NIK1/NIK1^G2048T^ HST6/HST6^A2926T^* | This study |
| CaLC3161  (resistant mutant #3) | As DPL15, *NIK1/NIK1^G1319C^ ORF19.2917/ORF19.2917^T401C^* | This study |
| CaLC3360  (resistant mutant #4) | As DPL15, *NIK1/NIK1^G218A^* *ORF19.4614/ORF19.4614^A639C^* | This study |
| CaLC3886 | As SN95, *YKE2/YKE2^T95A^* | This study |
| CaLC3841 | As SN95, *NIK1/NIK1^G2048T^* | This study |
| CaLC3872 | As DPL15, *NIK1/NIK1^G1319C^* | This study |
| CaLC2302  (SN250) | *his1*∆ */his1*∆ *leu2*∆*::C. dubliniensis HIS1 /leu2*∆*::C. maltosa LEU2 arg4*∆ */arg4*∆ *URA3/ura3 ::imm^434^ IRO1/iro1 ::imm^434^* | [3] |
| CaLC3942  (*nik1*∆*/nik1*∆*)* | *his1*∆*/his1*∆ *leu2*∆*/leu2*∆ *arg4*∆*/arg4*∆ *URA3/ura3*∆ *::imm^434^ IRO1/iro1 ::imm434 nik1*∆*:: C. dubliniensis HIS1/nik1*∆*:: C. maltosa LEU2* | [3] |
| CaLC3943  *(hog1*∆*/hog1*∆*)* | *his1*∆*/his1*∆ *leu2*∆*/leu2*∆ *arg4*∆*/arg4*∆ *URA3/ura3*∆ *::imm^434^ IRO1/iro1 ::imm434 hog1*∆*:: C. dubliniensis HIS1/hog1*∆*:: C. maltosa LEU2* | [3] |
| CaLC4199 | *his1*∆*/his1*∆ *leu2*∆*/leu2*∆ *arg4*∆*/arg4*∆ *URA3/ura3*∆ *::imm^434^ IRO1/iro1 ::imm^434^ hog1*∆*:: C. dubliniensis HIS1/hog1*∆*:: C. maltosa LEU2 NIK1/NIK1^G2048T^* | This study |
| CaLC3365  (CaSS1) | *ura3::imm^434^/ura3::imm^434^ his3::hisG/his3::hisG leu2::tetR-GAL4AD-URA/LEU2* | [4] |
| CaLC3465  (*tetO-ZRT1*/*zrt1*∆) | As CaSS1, *zrt1::HIS3/SAT1-tetp-ZRT1* | [4] |
| CaLC3464  (*tetO-ZRT2*/*zrt2*∆) | As CaSS1, *zrt2::HIS3/SAT1-tetp-ZRT2* | [4] |
| CaLC192  (BWP17) | *ura3::imm^434^/ura3::imm^434^ his1::hisG/ his1::hisG arg4::hisG/arg4::hisG* | [5] |
| CaLC2367 | As BWP17, *NRG1/NRG1*-3xHA-*ARG4* | This study |
| CaLC75 (CAI4) | *ura3::imm^434^/ura3::imm^434^* | [6] |
| CaLC564  (*ras1*∆*/ras1*∆*)* | As CAI4, *ras1::hisG/ras1::hph* | [7] |
| CaLC555  (*cyr1*∆*/cyr1*∆*)* | As CAI4, *cyr1::hisG-URA3-hisG/cyr1::hisG* | [8] |
| CaLC843  (*tpk2*∆/*tpk2*∆  tpk1∆/*PCK1p*-*TPK1*) | As CAI4, *URA3-PCK1p-TPK1/tpk1::hisG*  *tpk2::hisG/tpk2::hisG* | [9] |
| CaLC1900 (DAY286) | *ura3::imm^434^/ura3::imm^434^ ARG4::URA3::arg4::hisG/ arg4::hisG his1::hisG /his1::hisG* | [10] |
| CaLC4555 | As DAY286, *Tn-sok1/Tn-sok1* | [10] |
| CaLC4455  *(ubr1*∆*/ubr1*∆*)* | *his1*∆*/his1*∆ *leu2*∆*/leu2*∆ *arg4*∆*/arg4*∆ *URA3/ura3*∆ *::imm^434^ IRO1/iro1 ::imm^434^ ubr1*∆*:: C. dubliniensis HIS1/ubr1*∆*:: C. maltosa LEU2* | [3] |
| CaLC4530  *(ssk1*∆*/ssk1*∆*)* | *his1*∆ */his1*∆ *leu2*∆*::C. dubliniensis HIS1 /leu2*∆*::C. maltosa LEU2 arg4*∆ */arg4*∆ *URA3/ura3 ::imm434 IRO1/iro1 ::imm434 ssk1*∆*/ssk1*∆ | This study |
| CaLC3945  *(ssk2*∆*/ssk2*∆*)* | *his1*∆*/his1*∆ *leu2*∆*/leu2*∆ *arg4*∆*/arg4*∆ *URA3/ura3*∆ *::imm434 IRO1/iro1 ::imm434 ssk2*∆*:: C. dubliniensis HIS1/ssk2*∆*:: C. maltosa LEU2* | [3] |
| CaLC3947  *(pbs2*∆*/pbs2*∆*)* | *his1*∆*/his1*∆ *leu2*∆*/leu2*∆ *arg4*∆*/arg4*∆ *URA3/ura3*∆ *::imm434 IRO1/iro1 ::imm434 pbs2*∆*:: C. dubliniensis HIS1/pbs2∆:: C. maltosa LEU2* | [3] |
| CaLC2740 | *arg4*∆*/arg4*∆ *leu2*∆*/leu2*∆*:: C. albicans LEU2 his1*∆ */his1*∆*::C. albicans HIS1 URA3/ura3*∆*::imm434 IRO1/iro1*∆*::imm434* | [11] |
| CaLC2736  *(brg1*∆*/brg1*∆*)* | *arg4*∆*/arg4*∆ *leu2*∆*/leu2*∆ *his1*∆*/his1*∆ *URA3/ura3*∆*::imm434 IRO1/iro1*∆*::imm434*  *brg1*∆*::C. albicans LEU2/brg1*∆*:: C. albicans HIS1* | [11] |
| CaLC2738  *(rob1*∆*/rob1*∆*)* | *arg4*∆*/arg4*∆ *leu2*∆*/leu2*∆ *his1*∆*/his1*∆ *URA3/ura3*∆*::imm434 IRO1/iro1*∆*::imm434*  *rob1*∆*:: C. albicans LEU2/rob1*∆*:: C. albicans HIS1* | [11] |
| CaLC3433  *(efg1*∆*/efg1*∆*)* | *arg4*∆*/arg4*∆ *leu2*∆*/leu2*∆ *his1*∆*/his1*∆ *URA3/ura3*∆*::imm434 IRO1/iro1*∆*::imm434*  *efg1*∆*:: C. albicans LEU2/efg1*∆*:: C. albicans HIS1* | [11] |
| CaLC4453 | As SN250, *NRG1/NRG1-HA-ARG4* | This study |
| CaLC4454 | As CaLC4455, *NRG1/NRG1-HA-ARG4* | This study |

**S1 Table References**

1. Singh SD, Robbins N, Zaas AK, Schell WA, Perfect JR, Cowen LE. Hsp90 governs echinocandin resistance in the pathogenic yeast *Candida albicans* via calcineurin. PLoS Pathog. 2009;5: e1000532.

2. Noble SM, Johnson AD. Strains and strategies for large-scale gene deletion studies of the diploid human fungal pathogen *Candida albicans*. Eukaryot Cell. 2005;4: 298-309.

3. Noble SM, French S, Kohn LA, Chen V, Johnson AD. Systematic screens of a *Candida albicans* homozygous deletion library decouple morphogenetic switching and pathogenicity. Nat Genet. 2010;42: 590-8.

4. Roemer T, Jiang B, Davison J, Ketela T, Veillette K, Breton A, et al. Large-scale essential gene identification in *Candida albicans* and applications to antifungal drug discovery. Mol Microbiol. 2003;50: 167-81.

5. Wilson RB, Davis D, Mitchell AP. Rapid hypothesis testing with *Candida albicans* through gene disruption with short homology regions. J Bacteriol. 1999;181: 1868-74.

6. Fonzi WA, Irwin MY. Isogenic strain construction and gene mapping in *Candida albicans*. Genetics. 1993;134: 717-28.

7. Feng Q, Summers E, Guo B, Fink G. Ras signaling is required for serum-induced hyphal differentiation in *Candida albicans*. J Bacteriol. 1999;181: 6339-46.

8. Jain P, Akula I, Edlind T. Cyclic AMP signaling pathway modulates susceptibility of *Candida* species and *Saccharomyces cerevisiae* to antifungal azoles and other sterol biosynthesis inhibitors. Antimicrob Agents Chemother. 2003;47: 3195-201.

9. Bockmuhl DP, Krishnamurthy S, Gerads M, Sonneborn A, Ernst JF. Distinct and redundant roles of the two protein kinase A isoforms Tpk1p and Tpk2p in morphogenesis and growth of *Candida albicans*. Mol Microbiol. 2001;42: 1243-57.

10. Davis DA, Bruno VM, Loza L, Filler SG, Mitchell AP. *Candida albicans* Mds3p, a conserved regulator of pH responses and virulence identified through insertional mutagenesis. Genetics. 2002;162: 1573-81.

11. Homann OR, Dea J, Noble SM, Johnson AD. A phenotypic profile of the *Candida albicans* regulatory network. PLoS Genet. 2009;5: e1000783.
