# Supplementary material for: Metal Chelation as a Powerful Strategy to Probe Cellular Circuitry Governing Fungal Drug Resistance and Morphogenesis
Source: PLoS Genet. 2016 Oct 3;12(10):e1006350. doi: 10.1371/journal.pgen.1006350 (PMC5047589; doi:10.1371/journal.pgen.1006350)
Supplement: S2 Table — (DOCX) [file pgen.1006350.s010.docx]

**S2 Table.** Oligonucleotides used in this study.

**­­­­­­­­­­­­­­Primer Sequence (5’ 🡪 3’)**

| oLC243 | CAGGAAACAGCTATGAC |
| --- | --- |
| oLC274 | CTGTCAAGGAGGGTATTCTGG |
| oLC275 | AAAGTCAAAGTTCCAAGGGG |
| oLC752 | AGTATGTGGAGCTTTACTGGGA |
| oLC753 | CAGAAACACCAGCAACATCTTC |
| oLC2285 | GACCTTGAGATACCCAATTG |
| oLC2286 | CAGCTTGAATGGAAACGTAG |
| oLC2635 | CCACAATCAGTTTCTGCTTCTAC |
| oLC2636 | GACCTTGATGTGTTACAGATGG |
| oLC2637 | TGCTGCTTTGATAGCTTCTGG |
| oLC2638 | GGAGGGCAATTCCAGATAATTC |
| oLC3502 | TCCTTTCCTTTCCAATATAG |
| oLC3503 | CGGGGTACCAGACTTTATTCCTTCATTCC |
| oLC3504 | TTGCGGGCCCAGTACCCTGTATGAGGATAG |
| oLC3505 | TCCCCGCGGACTACTACTACTACTACCTG |
| oLC3506 | CCCGAGCTCTCTACTTGTAATATCATCCG |
| oLC3508 | CAGTAGTAGGACCTCCTTTG |
| oLC3509 | CGGGGTACCCCTCTCTGTTGTTTATCTCG |
| oLC3511 | AAAACGTTAGCCGTCAAAGC |
| oLC3512 | TTGCGGGCCCAAGTTACTACTGGGACCACATC |
| oLC3513 | TCCCCGCGGTAGTGACTGTTTAGGTTACTTG |
| oLC3514 | CCCGAGCTCTCATCTGCGAGGATGAGAAG |
| oLC3515 | TTGCGACATGGTAGTGTGCGTG |
| oLC3703 | TATTGCCAATGTCACCACTG |
| oLC3704 | AGAGGACAACCTCGTCAATC |
| oLC3785 | GTGCTTGGTATATTAGGAGG |
| oLC3786 | CTGAGTTGGCCAATAGTGCG |
| oLC3787 | GGTCCATCATTCTATTCCAC |
| oLC3788 | GCGTCAGCTTGAGTCAAAGC |
| oLC4151 | GGAGAATTTACAAGAACCCAG |
| oLC4152 | AGGTGAGCCCAATAATTCTG |
| oLC4240 | AACTTGTTGGGTCCTTTGGC |
| oLC4241 | TGCCTTGACTTGAGATCCC |
| oLC4370 | CAACAGACAACAACACAGGACTTTAGAAGCTTCACATGTTGGAACAAAATACAACACCAAGAGCCTAGTACCCGGGTACCCATACGATGT |
| oLC4371 | AAAAAACTAAACCCAAGCAATTAACCATCCAAATTTAACCCGTTTTATAATACAATTTTGACCACATCTATCGATGAATTCGAGCTCGTT |
| oLC4372 | AAAACACCAATGTCCATGGCC |
| oLC4373 | CAATGTAGCACCAAAAGGG |
| oLC4374 | TCATTACGACCGAGATTCCC |
| oLC4375 | GTGGTACTGCTAAAAGTGCC |
| oLC4427 | ACCAAGAAAGAAATAAAGAAACAAGAATTCTGCTTATAAAACGAATATAAAAAAAAAATAATAACTCATGGGAAACAGCTATGACCATG |
| oLC4428 | AAAACCGAAAACCTAATTTATTCCAACGACTCATCTTAGTGGCATTTCATAAATCCGTTTTTTTCTTTCAGTAAAACGACGGCCAG |
| oLC4429 | GTGCTCACTTTCTCATACTC |
| oLC4430 | GGATATTTGTTCACCACTGC |
| oLC4431 | GATACAGTAAACCTTCCCAC |
| oLC4581 | CTCTACTGGTGATTTACTGGCC |
| oLC4582 | GGCAGGACCATAAGATGGTTTC |
| oLC4583 | CACTTGCAACCCCAACAAACAC |
| oLC4584 | CTACTAAGCGTAATCTGGAACG |
| oLC3796 | GCTACTACTCCAAATACATC |
| oLC751 | AAGTGGATACTGTACCAGTTGG |
| oLC5109 | CTATGATGGATGCCATTCCC |
| oLC5115 | TCGACACAATGCTCAACACC |
| oLC5111 | TTGATAATCAGGGGCCACAC |
| oLC5112 | CGTTAACAGTTGTCCCCATC |
